# Recent Advances in the Design and Structural/Functional Regulations of Biomolecule‐Reinforced Graphene Materials for Bone Tissue Engineering Applications

**DOI:** 10.1002/smsc.202400414

**Published:** 2024-09-26

**Authors:** Dagang Li, Jinze Zhao, Yuan Wang, Jialu Wang, Zhenjuan Sun, Fuxin Wei, Gang Wei, Zhengang Sun

**Affiliations:** ^1^ Qingdao Huangdao Central Hospital Qingdao 266555 P. R. China; ^2^ The Affiliated Hospital of Qingdao University Qingdao 266000 P. R. China; ^3^ The Sixth People's Hospital of Qingdao Qingdao 266000 P. R. China; ^4^ Department of Orthopedics The Seventh Affiliated Hospital of Sun Yat‐sen University Shenzhen 518107 P. R. China; ^5^ College of Chemistry and Chemical Engineering Qingdao University Qingdao 266071 P. R. China

**Keywords:** biomolecules, bone engineering, graphene materials, structural regulation, two‐dimensional nanohybrids

## Abstract

Biomolecule‐reinforced graphene materials (Bio‐RGMs) have emerged as versatile matrices for biomedical and tissue engineering applications, owing to the combination of graphene‐based materials (GMs) with biomolecular components and their synergistic effects. In this review, an overview of the design, synthesis, structural/functional regulation, and bone engineering applications of various Bio‐RGMs is provided. Both covalent and noncovalent methods for conjugating biomolecules onto GMs, followed by an exploration of the structural diversity of Bio‐RGMs, ranging from 1D nanofibers to 2D membranes and 3D scaffolds/hydrogels/aerogels are discussed. Techniques such as electrospinning, self‐assembly, freeze‐drying, 3D printing, and templated synthesis are highlighted for their roles in designing and fabricating Bio‐RGM architectures. Additionally, specific properties and functions endowed to Bio‐RGMs by biomolecule conjugation, including biocompatibility, cytotoxicity, antibacterial activity, drug delivery ability, and fluorescent sensing are examined. Finally, recent advance is showcased in fabricating Bio‐RGMs for the bone tissue engineering applications of bone repair, regeneration, grafting, drug/cell delivery, and tumor inhibition, and further, the potential of Bio‐RGMs for preclinical applications is analyzed. It is believed that this review will deepen readers’ understanding of biomolecule–GM interactions and inspire the development of innovative Bio‐RGMs for advanced biomedical and tissue engineering applications.

## Graphical abstract

Biomolecule‐reinforced graphene materials (Bio‐RGMs) exhibit synergistic merits of biomolecules and graphene materials, revealing wide applications in various fields. This prehensive review focuses on the design and structural/functional regulations of Bio‐RGMs with high biocompatibility, cytotoxicity, antibacterial activity, controlled drug delivery, and fluorescent sensing, and further presents recent advances in the bioactive Bio‐RGMs for bone tissue engineering applications.

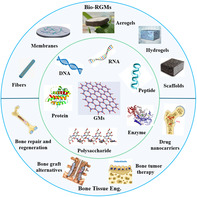

## 1 Introduction

Graphene‐based materials (GMs), including graphene oxide (GO), reduced graphene oxide (RGO), graphitic carbon nitride (g‐C_3_N_4_), and their derivatives, have exhibited widespread applications across various fields including materials science, nanotechnology, biosensors, energy storage, and environmental science.^[^ ^1^, ^2^, ^3^ ^]^ However, their relatively low biocompatibility, weak solubility, and potential cytotoxicity have constrained their extensive utilization in biomedicine and tissue engineering.^[^ ^4^, ^5^ ^]^ To overcome these limitations, considerable efforts have been directed toward enhancing the biological properties and functions of GMs.^[^ ^6^, ^7^ ^]^ Various chemical, physical, and biological treatments have been employed to adjust the active sites and functional groups of GMs,^[^ ^8^, ^9^, ^10^ ^]^ facilitating their conjugation with biological components. Despite these endeavors, the synthesis of GMs with desired biofunctions, such as high biocompatibility, biodegradability, and targeting capabilities, remains challenging. For instance, the biocompatibility and biodegradability of GO, RGO, and graphene quantum dots (GQDs) are very limited and their long‐term cytotoxicity to biological systems is unclear. Meanwhile, traditional GMs do have not the ability for specifically targeting with cells, which inhibits their potential applications in biomedicine and tissue engineering. In addition, it is not easy to achieve uniform and highly controllable functionalization of GMs via both chemical and physical modifications, which could induce undesired properties and unstable structure of GMs.

With the advancement of molecular nanotechnology, supramolecular chemistry, and biomimetic science, increasing attention has been focused on the design and synthesis of biomolecule‐reinforced graphene materials (Bio‐RGMs) for diverse applications.^[^ ^11^, ^12^, ^13^ ^]^ By leveraging both noncovalent and covalent interactions between GMs and biomolecules, the formed Bio‐RGMs can exhibit enhanced properties and synergistic new functions.^[^ ^14^, ^15^ ^]^ For instance, the conjugation of DNA aptamers,^[^ ^16^ ^]^ proteins,^[^ ^17^ ^]^ and antibodies^[^ ^18^ ^]^ onto GMs facilitates the development of Bio‐RGM‐based biosensors with heightened biorecognition, sensitivity, and selectivity, beneficial for biomedical diagnosis and trace substance monitoring. Bioengineering peptides with targeting abilities onto GMs enhances the targeting capability of the resulting peptide‐RGMs, offering promising applications in targeted drug delivery, cell bioimaging, and cancer therapy.^[^ ^19^, ^20^, ^21^ ^]^ Moreover, the synergistic biological and physical properties of biomolecules and GMs enable the fabrication of Bio‐RGMs with excellent mechanical strength, biocompatibility, and bioactivity, which are highly suitable for tissue engineering, regenerative medicine, and chemotherapy.^[^ ^22^, ^23^ ^]^

Recently, there has been a growing focus on harnessing the potential of Bio‐RGMs for bone and dental tissue engineering. Studies have shown that Bio‐RGMs possess the capability to act as a supportive matrix for bone tissue and facilitate the growth and regeneration of bone cells, owing to their favorable mechanical and biological properties.^[^ ^24^, ^25^ ^]^ The high specific surface area of GMs enables efficient loading of growth factors, drugs, and other biomolecules into bone tissues, thereby mediating the processes of bone growth and repair.^[^ ^23^, ^26^ ^]^ Additionally, electronic conductivity of GMs helps mimic the microenvironment of bone tissue, promoting the adhesion, growth, and proliferation of bone cells. In dental engineering, specially designed Bio‐RGMs serve as excellent components for tooth repair and cosmetology, as well as matrices for the synthesis of antibacterial dental materials.^[^ ^27^, ^28^ ^]^ For instance, Shuai et al. reported the design and synthesis of protein nanofibril (PNF)‐GO nanohybrids, which exhibited the functions to accelerate the cell adhesion and induce the osteogenic differentiation of stem cells.^[^ ^29^ ^]^ In another study, Wang et al. demonstrated the formed binding peptide‐hydroxyapatite (HA)‐graphene paper (GP) composites could simulate the osteogenic differentiation of stem cells and promote the expression of osteogenic biomarkers, verifying their promising application in bone tissue engineering (BTE) and biomedicine.^[^ ^30^ ^]^

Several important reviews on the synthesis of GMs for tissue engineering applications have been published.^[^ ^31^, ^32^, ^33^, ^34^, ^35^ ^]^ For example, Bai et al. provided a comprehensive review on the use of GMs as versatile platforms for nanotheranostics and tissue engineering.^[^ ^31^ ^]^ Their review focused on the applications of GMs in cardiac, nerve, bone, and skin tissue engineering. Similarly, Daneshmandi et al. demonstrated the applications of GMs for bone regenerative engineering,^[^ ^32^ ^]^ emphasizing the regulation of the biocompatibility and biodegradation of GMs. In a recent review, Edrisi et al. summarized the potential of GMs for cardiac tissue engineering,^[^ ^33^ ^]^ particularly discussing their role in angiogenesis, proliferation, and differentiation of stem cells. Based on the studies mentioned in the references, it becomes evident that highlighting the advancements in functional Bio‐RGMs for bone and dental tissue engineering would greatly enrich this promising research domain.

**Figure 1:**
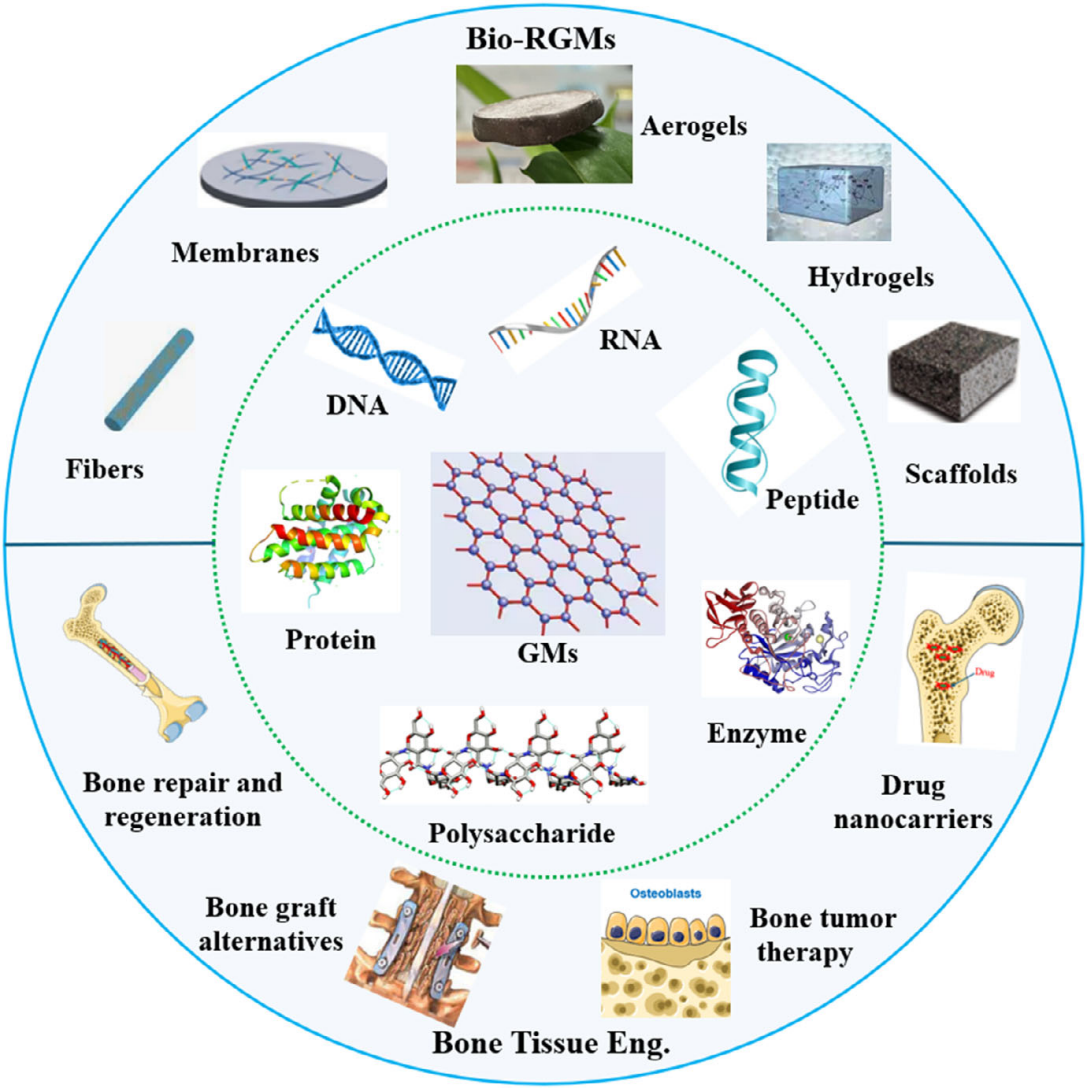
Schematic presentation on the construction of Bio‐RGMs for BTE applications.

Here, in this work, we present a progress report on the design, synthesis, and structural/functional regulations of various Bio‐RGMs for BTE applications, and the main contents are shown in **Figure** **1**. The first section (Section 1) introduces the research background of using Bio‐RGMs for various applications, followed by the presentation of the synthesis strategies of Bio‐RGMs through adjusting the biomolecule‐GM interactions (Section 2). Subsequently, we demonstrate structural regulation of Bio‐RGMs from 1D to 3D architectures through different fabrication techniques in Section 3. In Section 4, the strategies for improving the biological functions of Bio‐RGMs related to tissue engineering are further introduced and discussed. We then summarize recent studies on the use of Bio‐RGMs for BTE applications (Section 5). Finally, we provide our own viewpoints on the potential development and challenges of this promising topic. We believe that this review offers novel insights and significant contributions compared to previous reviews. Specifically, we provide detailed information on both the strategies for structural and functional tailoring of Bio‐RGMs and the structure‐efficiency relationships of designed Bio‐RGMs in BTE. It is anticipated that this work will advance the understanding of the interactions between biomolecules and GMs and facilitate the design of novel Bio‐RGMs for biomedical and tissue engineering applications.

## 2 Synthesis Methods of Bio‐RGMs via Biomolecule–GM Interactions

The interactions between biomolecules and material interfaces play a crucial role in facilitating the binding of biomolecules onto materials, leading to the formation of functional hybrids with multiple functions. These interactions encompass physical, chemical, and biological bindings occurring at atomic, molecular, and nanoscale levels. The strength and nature of biomolecule–material interactions significantly influence the biocompatibility, bioactivity, biorecognition, biotargeting, and bioadaptability of hybrid materials within biological systems. GMs serve as excellent platforms for investigating biomolecule–GM interactions, enabling the design and development of Bio‐RGMs with tailored functions and properties.^[^ ^12^ ^]^ Biomolecules attached onto the GM surface can enhance the bioreactivity, biotargeting, and biocompatibility of resulting Bio‐RGMs, thereby addressing the limitations of GMs and expanding their applications across various fields.

### 2.1 Covalent Biomolecule–GM Interactions for Bio‐RGMs

The conjugation of biomolecules, such as proteins, DNAs, enzymes, peptides, and others, onto GMs for the fabrication of Bio‐RGMs can be achieved via covalent biomolecule–GM interactions. These covalent interactions involve the formation of strong and stable chemical bonds (such as C—C, C—O, and C—N bonds) between GM surface and functional groups of targeted biomolecules. The formation of C—C bonds can be conducted by the reactions of cycloaddition or alkylation between GMs and molecules, and the C—O and C—N bonds can be formed through the reactions of the —OH or —COOH groups of GMs with epoxides or amino groups of targeted molecules. The covalent modification of GMs with biomolecules can change the chemical, mechanical, biological, and electronic properties of GMs, promoting their applications in bio‐related fields.

**Figure 2:**
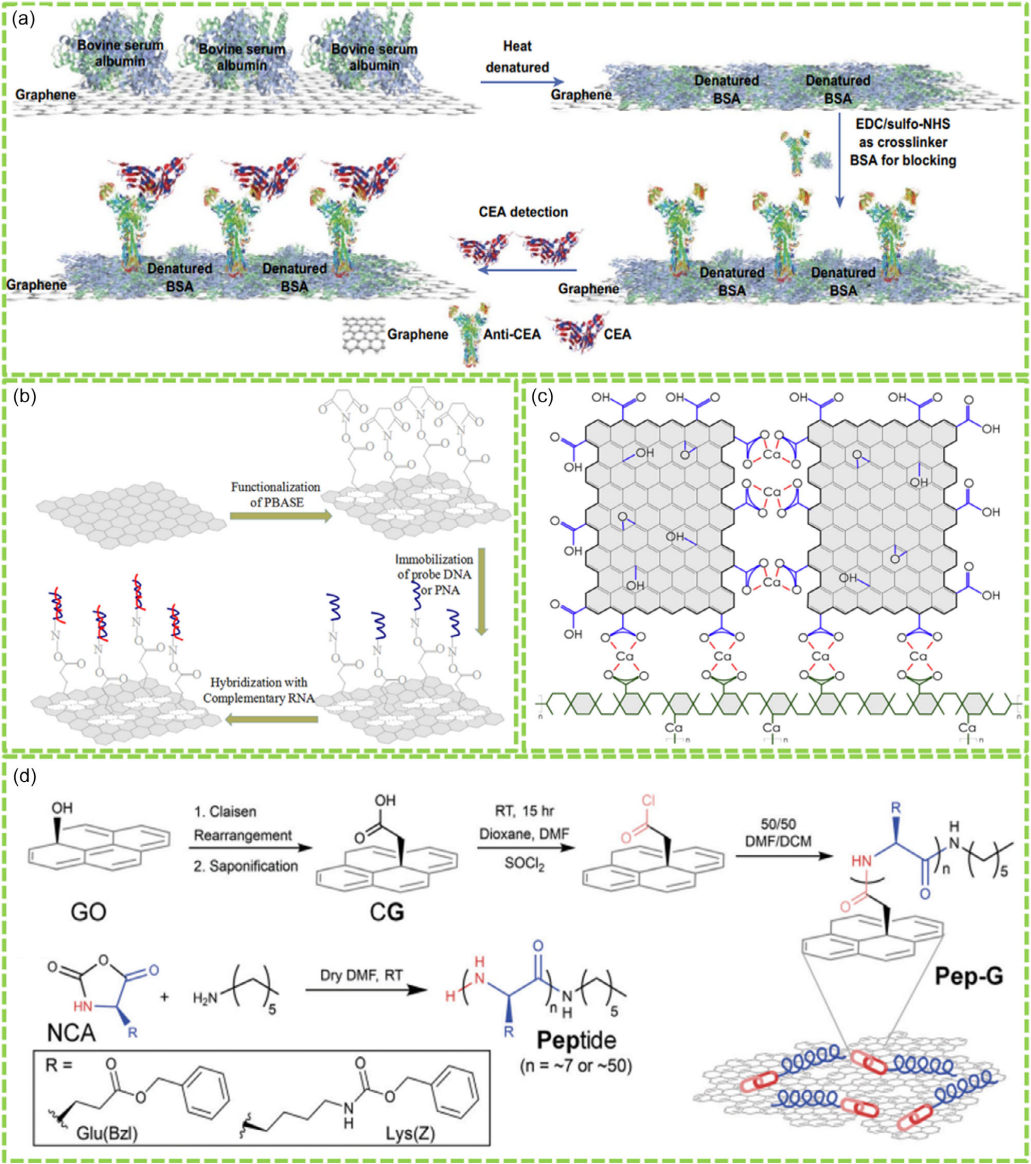
Design and synthesis of Bio‐RGMs via covalent biomolecule–GM interactions: a) EDC/NHS reaction for covalent binding anti‐CEA onto dBSA film. Reproduced with permission.^[^ ^40^ ^]^ Copyright 2019, Springer. b) Covalent binding DNA and RNA onto PBASE‐graphene composite. Reproduced with permission.^[^ ^42^ ^]^ Copyright 2020, Elsevier. c) Covalent binding peptides onto GO nanosheets. Reproduced with permission.^[^ ^43^ ^]^ Copyright 2019, Royal Society of Chemistry. d) Coordination reaction for alginate‐GO conjugates. Reproduced with permission.^[^ ^44^ ^]^ Copyright 2018, Elsevier.

As there are a large number of —OH and —COOH groups on the surface of GO, it was effective to modify the GO surface with *N‐*(3‐dimethylaminopropyl)‐*N*‐ethylcarbodiimide (EDC) and *N*‐hydroxysuccinimide (NHS), which can mediate the formation of GO‐NHS for covalent binding with the biomolecules with —NH— and —NH_2_ groups.^[^ ^36^ ^]^ This method was universal and has been utilized for the synthesis of a lot of Bio‐RGMs previously. For instance, Seelajaroen et al. achieved the immobilization of dehydrogenase (DH) enzymes on graphene for the synthesis of DH‐G nanohybrids as nanobiocatalysts.^[^ ^37^ ^]^ For example, Jagiello et al. demonstrated covalent modification of GO with Arg‐Gly‐Asp (RGD) peptide via the reaction of EDC· HCl/NHS with the amino groups of peptide.^[^ ^38^ ^]^ It was found that the conjugation of RGD peptide onto GO surface significantly enhanced its ability to bind L929 cell integrins, and did not show any effect on the viability of cells. With the same linker and method, Farrag et al. conducted the incorporation of antimicrobial peptides (AMPs, KRQRFYFRQRK, and QNKRFYFRKNQ) with RGO to form the peptide/RGO composites,^[^ ^39^ ^]^ which exhibited improved antibacterial effects toward *S. aureus* and *P. aeruginosa*. The improved antibacterial performance of the formed peptide/RGO composites was ascribed to the sharp edge structure of RGO and the unique antibacterial ability of AMPs. In another case, Zhou et al. reported the synthesis of multifunctional Bio‐RGMs by immobilizing both bovine serum albumin (BSA) and anticarcinoembryonic antigen (anti‐CEA) via noncovalent and covalent interactions, respectively.^[^ ^40^ ^]^ As shown in **Figure** **2**a, denatured BSA (dBSA) molecules were bound onto the chemical vapor deposition (CVD)‐prepared graphene film via *π*–*π* stacking. After that, anti‐CEA antibodies were linked onto the dBSA film via the EDC and sulfo‐NHS reaction, resulting anti‐CEA‐dBSA‐graphene film. The fabricated multifunctional Bio‐RGM film was further used as a graphene field‐effect transistor (FET) biosensor for highly selective and sensitive detection of CEA. This study indicates that the nano‐dBSA modification strategy created new function for the formed dBSA‐G sensor platform, showing great importance for promoting GMs in clinical diagnosis and drug delivery.

In addition, covalent binding biomolecules onto GMs can be achieved using bifunctional molecular linkers with benzene ring and ‐NHS residue on both sides. Previously, Wei et al. reported the noncovalent modification of RGO with 1‐pyrenebutyric acid *N*‐hydroxysuccinimide ester (PNHS) for the synthesis of ferritin‐RGO nanohybrids.^[^ ^41^ ^]^ In this case, the pyrenebutyric group mediated the binding with graphene surface via *π*–*π* interaction and the NHS group promoted the conjugation with the amino group of ferritin protein through covalent reaction. The created ferritin‐RGO promoted biomimetic synthesis of functional RGO‐FePt nanohybrids with high solubility, ferromagnetism, and electrocatalytic activity. This technique is facile and effective and can be used for the conjugation of many biomolecules, such as protein, peptide, enzymes, and amino‐modified DNA/RNA, onto GMs for the fabrication of versatile Bio‐RGMs. In another case, Tian et al. presented the covalent immobilization of peptide nucleic acid (PNA) and DNA probes onto graphene for the fabrication of graphene FET biosensors for detecting RNA.^[^ ^42^ ^]^ As depicted in Figure 2b, CVD‐created graphene is functionalized with 1‐pyrenebutanoic acid succinimidyl ester (PBASE), which serves as a bifunctional linker and provides *π*–*π* interaction with graphene surface with one end and another end (‐NHS) to interact with amino‐modified DNA and PNA probes. Therefore, both probes could be immobilized onto the graphene surface covalently. This Bio‐RGM platform was selective to detect the target RNA molecules with high sensitivity.

The conjugation of bioactive peptides onto GMs has been achieved with a novel covalent strategy.^[^ ^43^ ^]^ As shown in Figure 2c, the —OH group of GO is transferred to —COOH by a Claisen rearrangement through a *sp* ^3^ methylene bond, which is then reacted with SOCl_2_ to form acyl chloride. Finally, *N*‐carboxyanhydride (NCA)‐protected bioactive peptides are conjugated onto GO through a controlled ring‐open polymerization, creating the Pep‐G conjugates. In addition, the Pep‐G conjugates formed uniform nanocoating via self‐assembly, which exhibited high cytocompatibility and electronic conductivity, promoting their potential application in regenerative engineering. This covalent functionalization technique is effective and can be utilized to create a lot of cell‐instructive surfaces for biomedical applications.

Coordination interaction is also a potential force to promote the formation of Bio‐RGMSs by introducing cations as the bridges for GMs and biomolecules. For instance, Serrano‐Aroca et al. reported a green synthesis strategy of alginate‐GO (ALG‐GO) composite hydrogels through Ca^2+^‐induced coordination interaction.^[^ ^44^ ^]^ As shown in Figure 2d, Ca^2+^ can mediate the coordination reactions between GO and alginate chains, promoting the formation of stable network structure. This technique is useful for the formation of polysaccharide‐reinforced GMs via the coordination chemical reactions. In addition, it is facile and green, and could be utilized for the large‐scale production of ALG‐GO composite hydrogels. The introduction of ALG onto GO surface greatly enhanced physical properties of the ALG‐GO composites, including improved thermal properties and wettability, ascribing to the reorganization of the novel hybrid structure.

### 2.2 Noncovalent Biomolecule–GM Interactions for Bio‐RGMs

Besides, through noncovalent interactions between biomolecules and GMs many synthesis strategies have been widely used for the preparation of Bio‐RGMs. These noncovalent interactions include electrostatic interactions, *π*–*π* stacking, van de Waals force, hydrogen bond, and others. The electrostatic interactions are based on the force between charged molecules or ions with the charges on GM surface, and the *π*–*π* stacking can be formed through the interactions between the *π* electrons of GMs and aromatic molecules or DNA bases. In addition, the weak noncovalent interactions, like van de Waals force and hydrogen bond, are also important for promoting the applications of GMs in gas adsorption, sensors, molecular recognition, and drug nanocarriers.

**Figure 3:**
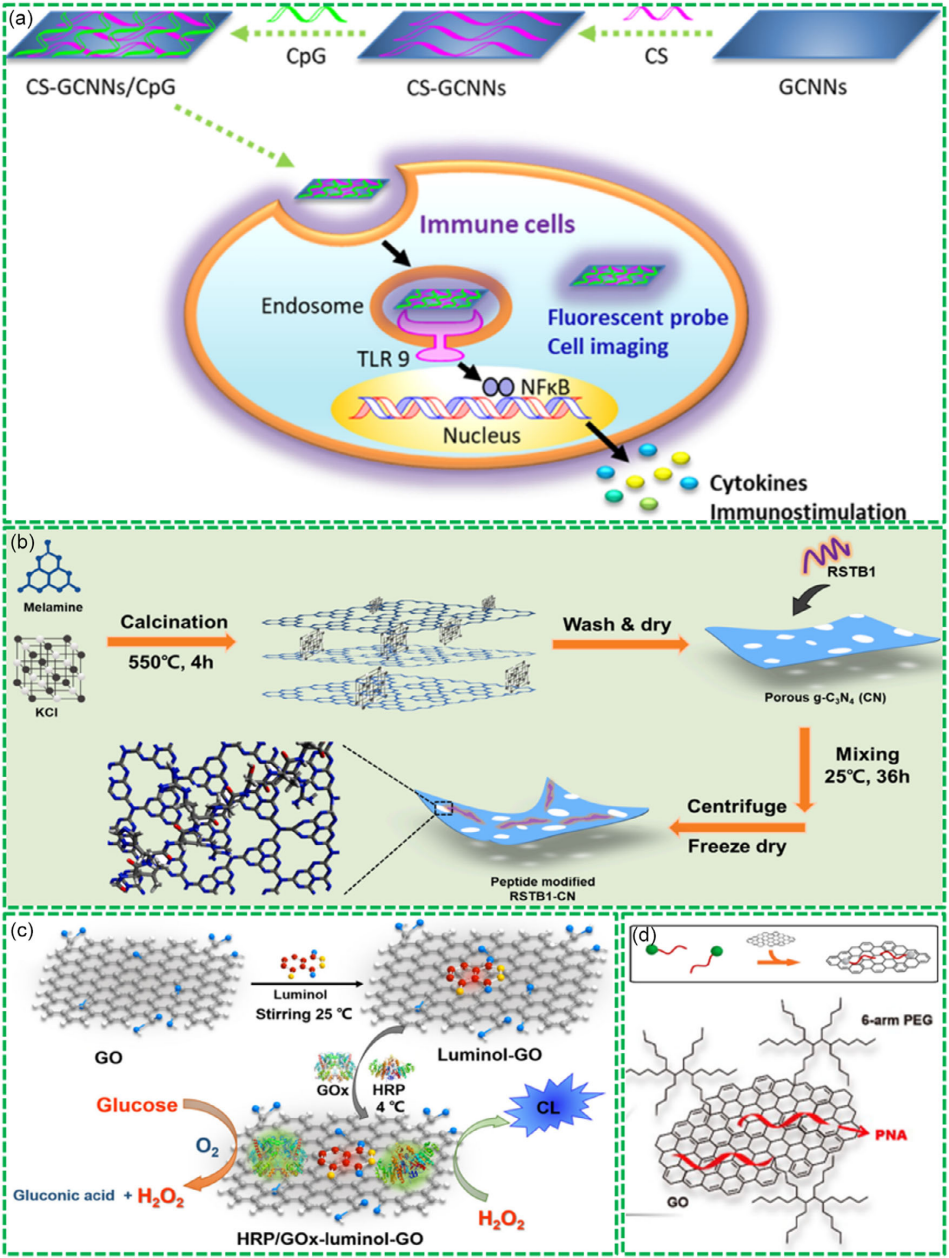
Design and synthesis of Bio‐RGMs via noncovalent biomolecule–GM interactions: a) CS‐GCNNs composite formed via electrostatic interactions for CpGODN delivery and imaging. Reproduced with permission.^[^ ^48^ ^]^ Copyright 2021, American Chemical Society. b) RSTB1‐GCNNs composite formed via electrostatic interactions. Reproduced with permission.^[^ ^49^ ^]^ Copyright 2024, Elsevier. c) Modification of GO with luminol via *π*–*π* stacking. Reproduced with permission.^[^ ^52^ ^]^ Copyright 2018, Springer. d) Formation of GO‐PNA via *π*–*π* stacking. Reproduced with permission.^[^ ^55^ ^]^ Copyright 2018, American Chemical Society.

Polymers are excellent components for the coating of GMs with positive and negative charges, which then mediate the electrostatic adsorption with biomolecules under adjusting the pH value of solution systems, to form Bio‐RGMs. For instance, previously Wang et al. demonstrated the coating of RGO with poly(diallyldimethylammonium chloride) (PDDA) to form positively charged PDDA‐RGO composites, which exhibited electrostatic binding with self‐assembled negatively charged PNFs to form PDDA‐RGO/PNF composites.^[^ ^45^ ^]^ The formed PDDA‐RGO/PNF hybrids have been applied for the fabrication of electrochemical biosensors with enhanced sensing performance. In another cases, through the self‐polymerization process, polydopamine (PDA) was coated onto GO surface for the synthesis of PDA@GO nanocomposites for biomimetic synthesis of functional hybrid nanomaterials.^[^ ^46^, ^47^ ^]^ Besides GO and RGO, g‐C_3_N_4_ has also been functionalized with biomolecules, such as chitosan (CS) and peptides, for the synthesis of g‐C_3_N_4_‐based composite materials via electrostatic interactions. For instance, Zhang et al. reported electrostatic modification of g‐C_3_N_4_ nanosheets (GCNNs) with CS for bioimaging and drug delivery applications.^[^ ^48^ ^]^ As shown in **Figure** **3**a, the as‐prepared GCNNs were negatively charged in PBS solution, ascribing to the adsorption of SO_4_^2−^ ions in the synthesis process. Therefore, cationic CS can be bound onto the GCNN surface through electrostatic interactions. After further binding CpG oligodeoxynucleotides (ODNs), the formed CS‐GCNNs‐CpGODN could be used for tracing the process of CpGODN delivery with direct cell imaging. This study presents an inspired study on the synthesis of GCNN‐based Bio‐RGMs as an excellent nanoplatform for cancer immunotherapy. In a recent study, Wu et al. demonstrated the modification of GCNNs with peptides via electrostatic interactions between the amine groups of peptides (RSTB1) and the edge N‐atoms of GCNNs,^[^ ^49^ ^]^ as shown in Figure 3b. Due to the unique interactions between RSTB1 and GCNNs, the electronic structure of GCNNs was modulated to obtain enhanced visible light absorption, creating 14‐time higher photocatalytic H_2_ production than pristine GCNNs. This study makes it possible to design and synthesis of metal‐free Bio‐RGM photocatalysts for high‐performance production of H_2_.

Utilizing the *π*–*π* interactions between the benzene groups of biomolecules and the aromatic carbon rings of GMs, it is facile to form biocompatible Bio‐RGMs through simple mixing and quick conjugation. For instance, histidine‐rich peptides have been reported to interact with the graphene surface via *π*–*π* stacking and hydrophobic interactions,^[^ ^50^ ^]^ and the peptides with the motif of YWYAF have been widely used for the design and synthesis of functional Bio‐RGMs for biosensor and cancer therapy applications.^[^ ^20^, ^51^ ^]^ It was found that the peptides with graphene‐binding motifs showed specifically strong interactions with the graphene surface, showing high advantages for the formation of biocompatible and bioactive Bio‐RGMs for biomedical and tissue engineering applications. Besides peptides, it has been reported that some biomolecules with benzene rings could be bound onto the graphene surface through *π*–*π* interactions for the modification of GMs and the formation of functional Bio‐RGMs.^[^ ^52^ ^]^ As shown in Figure 3c, the luminol molecules with potential chemiluminescence property were adsorbed onto GO effectively via *π*–*π* interactions. After simple mixing under stirring, functional luminol‐GO hybrids could be created effectively, which served as a good nanoplatform for the immobilization of enzymes of horseradish peroxidase and glucose oxidase (GOx) for the fabrication of chemiluminescence glucose biosensors. The fabricated glucose biosensors exhibited a low detection limit of 1.2 nm with a wide linear detection range of 5.0–5.0 mm, presenting high potential for the trace‐detection of glucose in human blood samples.

In addition, ssDNA, dsDNA, and PNA molecules have shown potential *π*–*π* interactions with GMs.^[^ ^53^ ^]^ Husale et al. investigated the interactions between both ssDNA and dsDNA with graphene surface with atomic force microscopy, and identified that ssDNA bound with graphene and the corresponding *π*–*π* stacking interactions were quantified.^[^ ^54^ ^]^ As demonstrated in Figure 3d, PNA molecules are conjugated noncovalently onto GO surface for the formation of GO‐PNA composites.^[^ ^55^ ^]^ The synthesized PNA and GO‐based Bio‐RGMs have been applied as drug nanocarriers for cancer therapy previously.

### 2.3 Summary of Covalent and Noncovalent Interactions

In this section, we introduce various covalent and noncovalent interactions between biomolecules and GMs briefly, which show the conjugation mechanism from the viewpoints of molecular level and molecule‐material interface interactions. The readers are suggested to refer to our previous review to get more details on the biomolecule–GM interactions.^[^ ^12^ ^]^

These interactions play crucial roles in the design and synthesis of functional Bio‐RGMs for specific applications. However, their advantages and disadvantages should be also considered for optimal synthesis of Bio‐RGMs. Covalent interactions of GMs have the advantages of enhanced chemical stability and functionability, flexible chemical reactions, and controllable modification density. Meanwhile, they would affect the *π*‐system of GMs via destroying the *sp* ^2^ hybrid structure and inducing surface defects, resulting in reduced electronic performance. In addition, covalent modifications of GMS usually need complex reaction processes, harsh reaction conditions, and using of toxic chemicals, which could potentially cause the problems of environmental and biological safety.

Noncovalent synthesis of Bio‐RGMs via physical adsorption and weak molecule‐material interactions does not involve in the formation of chemical bonds, and will not alter original structure and electronic and mechanical properties of GMs. The functionalization process is simple, cost‐effective, and environmental friendly. Their shortcomings are also obvious. First, the formed Bio‐RGMs show low stability under some conditions such as specific pH, temperature, and ionic strength due to the weak conjugation between biomolecules and GMs. Second, the degree of modification is limited. It is hard to achieve the functionalization of GMs with high density via noncovalent interactions, which will result in low application performance and selectivity.

In both types of synthesis methods toward Bio‐RGMs, the synthesis processes could greatly affect the structure and properties of final Bio‐RGMs, as well as their BTE applications. First, the adjustment of the size, morphology, and dimension of Bio‐RGMs can affect the adhesion of bone cells. In this case, the construction of 3D porous structure that is similar to the ECM structure will be beneficial for the growth and differentiation of bone‐related cells. Second, the functionalization degree of GMs via both covalent and noncovalent interactions is crucial for the adsorption of biomolecules. The formation of Bio‐RGM with high‐dense biomolecular binding is useful for mediating the biomineralization process and the formation of apatite for bone repair. Third, the doping of inorganic nanoparticles, such as CaO, MgO, GNPs, and others into Bio‐RGMs can also extend their BTE applications.

To make it more clear, here, we provide a **Table** **1** to summarize the covalent and noncovalent interactions between GMs and various biomolecules, as well as the chemicals for mediating the interactions and specific applications of the formed Bio‐RGMs.

**Table 1:** Summary of the interactions, GMs, chemicals, biomolecules, and applications of designed Bio‐RGMs.

Interactions	GMs	Chemicals	Biomolecules	Application	References
	GO	Mannosylated ethylenediamine	D‐mannose	Hemolysis	[36]
	Graphene	EDC/S‐NHS	DH	Biocatalysts	[37]
	GO	EDC‐HCl/NHS	RGD	Cell culture	[38]
	RGO	EDC‐HCl/NHS	AMPs	Antibacteria	[39]
Covalent interactions	Graphene	EDC/S‐NHS	Anti‐CEA	FET biosensors	[40]
	RGO	PNHS	Ferritin	Electrocatalysis	[41]
	Graphene	PBASE	PNA/DNA	Biosensors	[42]
	RGO	SOCl2/NCA	Peptide	Cell culture	[43]
	GO	CaCl2	Alginate	Tissue engineering	[44]
	RGO	PDDA	PNFs	Biosensors	[45]
	GO	–	PDA	Catalysis	[46, 47]
	GCNN	–	CS/ODNs	Drug delivery	[48]
Noncovalent interactions	GCNN	–	RSTB1	Photocatalysis	[49]
	Graphene	–	H‐rich peptide	Cell culture and drug carrier	[50]
	GO	–	YWYAF‐peptide	Biosensors	[51]
	GO	–	Luminol	Enzyme immobilization	[52]
	Graphene	–	ssDNA/dsDNA	Biosensors	[54]
	GO	–	PNA	Drug nanocarriers	[55]

## 3 Techniques of Structural Regulations for Bio‐RGMs

Bio‐RGMs have demonstrated significant potential across various applications, including tissue engineering and regeneration, antibacterial materials, drug delivery, biosensing, and bioimaging. These applications are intricately linked to the structures and functionalities of designed Bio‐RGMs. In this section, we analyze the structural modulation of Bio‐RGMs, transitioning from 1D nanofibers and 2D membranes to 3D scaffolds, aerogels, and hydrogels. To construct functional nanostructures based on Bio‐RGMs for biomedical applications, various techniques, including electrospinning, vacuum‐assisted filtering, templated synthesis, self‐assembly, freeze‐drying, and 3D printing, have been employed. In this section, we present these fabrication techniques for tailoring the structure of Bio‐RGMs briefly.

**Figure 4:**
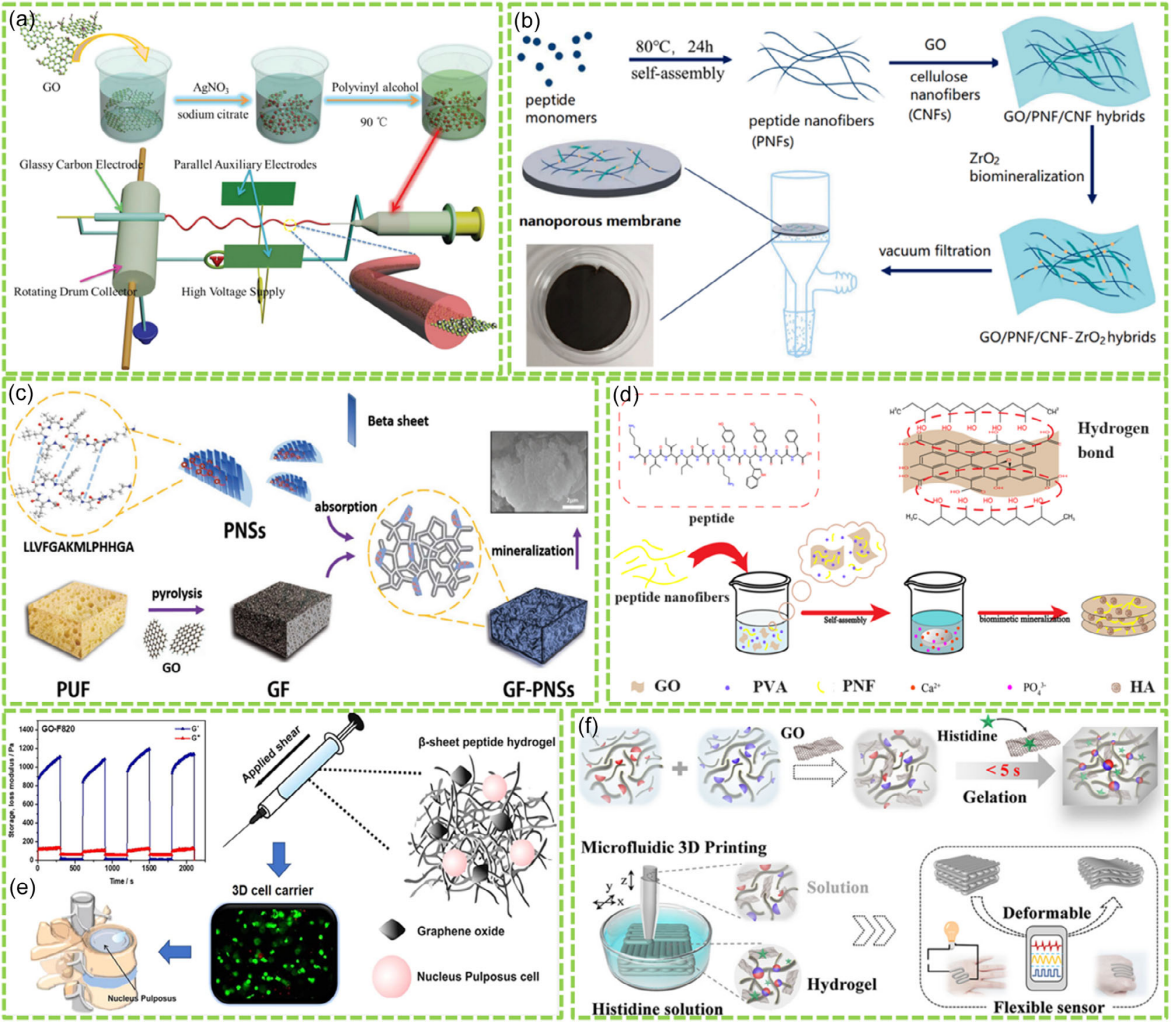
Construction of Bio‐RGMs into 1D, 2D, and 3D structures: a) electrospun nanofibers. Reproduced with permission.^[^ ^56^ ^]^ Copyright 2016, Wiley. b) Vacuum‐assisted filtration created membranes. Reproduced with permission.^[^ ^62^ ^]^ Copyright 2022, Elsevier. c) Templated scaffolds. Reproduced with permission.^[^ ^64^ ^]^ Copyright 2018, Wiley. d) Self‐assembled aerogels by freeze‐drying. Reproduced with permission.^[^ ^67^ ^]^ Copyright 2022, Royal Society of Chemistry. e) Self‐assembled hydrogels. Reproduced with permission.^[^ ^70^ ^]^ Copyright 2019, Elsevier. f) 3D‐printed hydrogels. Reproduced with permission.^[^ ^71^ ^]^ Copyright 2022, American Chemical Society.

Electrospinning is a facile technique for the preparation of polymer‐based nanofibers and membranes. By introducing GMs or Bio‐RGMs into the electrospun polymer nanofibers, the interactions between polymer molecules and GMs or Bio‐RGMs can mediate the formation of GM‐ or Bio‐RGM‐based nanofibers. For instance, Li et al. reported the preparation of polyvinyl alcohol (PVA) nanofibers decorated with GO and AgNPs by electrospinning, as shown in **Figure** **4**a.^[^ ^56^ ^]^ After incorporating GO nanosheets, the formed PVA‐GO‐AgNPs hybrid nanofibers exhibited a stable structure, high mechanical strength, and improved biochemical functionality, suggesting potential applications in the construction of electrochemical biosensors. Through electrospinning, the assembly of porous g‐C_3_N_4_ nanosheets into PCL nanofibers for BTE application has been achieved.^[^ ^57^ ^]^ The introduction of g‐C_3_N_4_ into PCL nanofibers decreased the diameter of electrospun fibers, and enhanced material mechanical properties, biocompatibility, and biodegradability. Therefore, the created PCL/g‐C_3_N_4_ composites could serve as new platform for BTE application potentially.

In a recent study, Budi et al. demonstrated the fabrication of Gel/PCL nanofibers reinforced by dicalcium phosphate‐modified GO (DPmGO) using a co‐electrospinning method.^[^ ^58^ ^]^ The obtained results indicated that the created Gel/PCL‐DPmGO hybrid nanofibers had high biocompatibility, high hydrophilicity, and good antibacterial activity. These properties suggest their potential utility in BTE.

2D Bio‐RGM‐based membranes and films are potential nanoplatforms for the cell adhesion and proliferation in tissue engineering field. It is well known that functional membranes and films can also be prepared by the electrospinning technique. By electrospinning, GM‐based functional membranes, including the GO‐PLGA,^[^ ^59^ ^]^ GO/CS/HA,^[^ ^60^ ^]^ and PDA‐PU‐GO^[^ ^61^ ^]^ nanofibrous membranes have been prepared for BTE applications. In addition, the vacuum‐assisted filtration technique has been applied for the construction of Bio‐RGM‐based membranes. As shown in Figure 4b, both cellulose nanofibers (CNFs) and self‐assembled PNFs were bound onto GO nanosheets for the formation of PNF/CNF/GO nanohybrids, which further mediated the formation of ZrO_2_ NPs via biomimetic mineralization.^[^ ^62^ ^]^ The peptide (YYACAYY) showed noncovalent interactions (such as *π*–*π* interaction and hydrogen bond) with GO surface, and the CNFs were further chemically modified to interact with GO through covalent binding. The formed PNF/CNG/GO‐ZrO_2_ nanohybrids were vacuum‐filtrated to fabricate PNF/CNG/GO‐ZrO_2_ nanohybrid membranes. This technique is facile, economical, and effective, and can be applied to prepare various bioactive membranes for cell culture and bone growth. In a recent study, He et al. fabricated PNF/GO/AgNPs hybrid membranes with high biocompatibility and antibacterial properties through an evaporation‐induced interface self‐assembly process.^[^ ^63^ ^]^ The formed PNF/GO/AgNPs hybrid membrane exhibited potential application for wound dressing and healing ascribing to the multiple functions of the membranes.

Besides 1D nanofibers and 2D membranes, 3D structures such as scaffolds, aerogels, and hydrogels based on Bio‐RGMs have also been constructed through several fabrication strategies. As shown in Figure 4c, Li et al. demonstrated the template synthesis of graphene foam‐peptide nanosheets (PNSs) scaffolds.^[^ ^64^ ^]^ The peptides, designed with specific motifs, self‐assembled into PNSs, exhibiting high affinity to the graphene surface via *π*–*π* interactions. Consequently, 3D scaffolds composed of GF‐PNSs were fabricated through template synthesis and PNS binding, offering potential application of biomineralized Bio‐RGM scaffolds in BTE, capitalizing on the biomimetic properties of peptides, and 3D porous structure of materials. In addition to the templated synthesis methods and self‐assembly, electrospinning, and 3D printing have also been utilized for the fabrication of Bio‐RGM scaffolds for BTE applications. For instance, Ali et al. reported the fabrication of electrospun nanofibrous scaffolds based on graphene nanosheets (GNs)/CS/gelatin nanocomposites, exhibiting high antimicrobial and wound healing activities.^[^ ^65^ ^]^ The results indicated that the diameter and porosity of the electrospun nanofibers can be regulated by the content of GNs. By the reinforcement of 0.15% GNs, the formed hybrid nanofibers exhibited the least diameter of 106 ± 30 nm and the highest porosity of about 90%. Similarly, Wang et al. reported the construction of PCL/G scaffolds using 3D printing for BTE.^[^ ^66^ ^]^ They indicated that these designed scaffolds, coupled with electrical stimulation, could effectively mediate bone regeneration.

Bio‐RGMs can also be constructed into 3D aerogels through freeze‐drying the formed prescaffolds and hydrogels. Yang et al. reported the synthesis of PNF‐functionalized GO for the biomineralization of HA.^[^ ^67^ ^]^ After peptide self‐assembly and the formation of composite PVA hydrogels, freeze‐drying was applied to construct PNF/GO/HA/PVA aerogels. These aerogels, as shown in Figure 4d, could be utilized for both environmental water purification and tissue engineering purposes. Asha et al. reported the preparation of CS‐reinforced RGO aerogels by freeze‐drying, which could further induce the biomineralization of HA for bone tissue regeneration.^[^ ^68^ ^]^ In a subsequent study, they demonstrated that various polymer‐reinforced GO aerogels, such as collagen (COL)‐GO, CS‐GO, and PLGA‐GO, could serve as excellent matrices for the biomineralization of HA for BTE applications.^[^ ^69^ ^]^

Hydrogels based on Bio‐RGMs are excellent matrices for biomedical and tissue engineering applications due to their high biocompatibility, mechanical stability, adjustable shape, and high specific surface area. These hydrogels can be prepared through chemical or physical cross‐linking, self‐assembly, and advanced techniques such as 3D printing and electrospinning. For instance, Ligorio et al. demonstrated the preparation of PNF hydrogels containing GO via electrostatic and hydrophobic interactions between FEFKFEFK peptide and GO nanosheets (Figure 4e).^[^ ^70^ ^]^ The created PNF/GO hybrid hydrogels exhibited high cell viability and metabolic activity, indicating their potential for delivering nucleus pulposus for intervertebral disc repair. In another case, Ding et al. reported the construction of GO/peptide hybrid hydrogels through histidine‐assisted microfluidic 3D printing technique,^[^ ^71^ ^]^ as shown in Figure 4f. The formed 3D GO/peptide hybrid hydrogels exhibited excellent conductivity and could be applied for the fabrication of wearable sensors to monitor the motion changes. Zhang et al. reported the preparation of RGO/GelMA hybrid hydrogels by 3D printing.^[^ ^72^ ^]^ It was found that these 3D‐printed hydrogels exhibited low inflammatory response and promoted nerve and bone growth, suggesting potential applications in various areas of biomedicine and tissue engineering.

Based on the introduction and discussion presented above, it is evident that the structures of Bio‐RGMs can be tailored using various techniques from nanocomposites to 1D, 2D, and 3D forms. This structural versatility has significantly enhanced the practical applications of Bio‐RGMs in tissue engineering and other fields. To make it more clear, here we summarize the abovementioned techniques for structural regulation of Bio‐RGMs, and indicate their advantages and shortcomings, as shown in **Table** **2**.

**Table 2:** Summary of the techniques for structural regulation of Bio‐RGMs.

Techniques	Bio‐RGMs	Structure	References	Advantages	Shortcomings
Electrospinning	PVA‐GO‐AgNPs	Nanofibers	[56]	Simple, cost‐effective, controllable, suitable for various materials, and wide applications	Low production, complex process, and aggregation of nanofibers
	g‐C3N4‐PCL	Nanofibers	[57]		
	GO‐Gel/PCL	Nanofibers	[58]		
	GO‐PLGA	Membrane	[59]		
	GO/CS/HA	Membrane	[60]		
	PDA‐PU‐GO	Membrane	[61]		
	GNs/CS/GEL	3D scaffolds	[65]		
Vacuum‐assisted filtration	PNF/CNF/GO	Membrane	[62]	Operation simple, large‐size membrane, and quick production	Special filter needed, block of the filter pores, and sensitive to conditions
Self‐assembly	PNF/GO/AgNPs	Membrane	[63]	Low cost, gentle conditions, simple, structure‐controllable	Low stability, possible defects, and required self‐assembly conditions
	PNF‐GO	Hydrogels	[70]		
Templated synthesis	GF‐PNSs	3D scaffolds	[64]	Controlled structure, adjustable size and pores, and high reproducity	High cost, need to remove the template, and structure defects
3D printing	PCL/G	3D scaffolds	[66]	Uniform structure, flexible, and designable	High cost, slow production, and limited ink materials
	GO/peptide	Hydrogels	[71]		
	RGO/GelMA	Hydrogels			
Freeze‐drying	PNF/GO/HA/PVA	Aerogels	[67]	Porous materials, high stability, suitable for many materials	High cost and long operation time
	CS‐RGO	Aerogels	[68]		
	COL‐GO, CS‐GO, PLGA‐GO	Aerogels	[69]		

## 4 Specific Properties and Functions of Bio‐RGMs

Graphene and its derivatives boast several advantages, including a large specific surface area, excellent electrical conductivity, ideal optical properties, satisfied mechanical strength, availability for mass production, and easy modification.^[^ ^73^, ^74^ ^]^ These characteristics render them highly promising for biomedical and tissue engineering applications. Commonly used modification materials to create Bio‐RGMs include metals, metal oxides (MOs), quantum dots (QDs), polymers, and bioactive molecules, which could enhance the stability and dispersion of Bio‐RGMs in various solution conditions, ensuring their biosafety, biocompatibility, and processability.^[^ ^75^, ^76^ ^]^ A previous study demonstrated that loading curcumin (Cur) onto GO nanosheets reduced its toxicity toward human bronchial epithelial cells while exhibiting anticancer effects.^[^ ^77^ ^]^ In this section, we highlight the unique properties of various types of Bio‐RGMs.

### 4.1 Biocompatibility and Cytotoxicity

Biomaterials intended for biomedicine and tissue regeneration must possess excellent biocompatibility, biological activity, tissue‐inducing capability, and adequate physico‐mechanical performance. Functionalized GMs have garnered significant attention for their potential applications in tissue engineering. It is widely acknowledged that the prerequisite for tissue regeneration is the ability to guide the proliferation and controlled differentiation of stem cells. Recent studies have underscored the significant impact of GMs on the regeneration of various tissues, including nerves, bones, cartilage, skeletal muscles, hearts, skin, and adipose tissue, as well as their effects on stem cells.^[^ ^78^ ^]^ Bio‐RGMs have been shown to effectively enhance the adhesion, proliferation, and differentiation of various mammalian stem cells.

The mechanisms underlying the promotion of tissue regeneration by Bio‐RGMs are still under investigation. For instance, in the context of soft tissue repair, Bio‐RGMs may serve as biological scaffolds or templates, offering structural support for specific cells and facilitating the formation of new tissues.^[^ ^79^ ^]^ Augmented tissue guidance could be achieved more effectively by incorporating inducers or growth factors. In bone engineering, Bio‐RGMs have been observed to exert a beneficial effect on bone regeneration by promoting osteogenic differentiation and maturation toward an osteoblastic phenotype, which was attributed to various mechanisms, including increased mineral deposition, activation of key gene expression, and enhancement of protein production involved in bone formation.^[^ ^80^ ^]^ The application of Bio‐RGMs in bone engineering will be comprehensively reviewed in Section 5.

Compared to pristine graphene, GO nanosheets feature a plethora of oxygenated functional groups on their surface, endowing them with physiological stability, high hydrophilicity, and excellent solubility and dispersibility in aqueous solutions.^[^ ^81^, ^82^, ^83^ ^]^ These characteristics make GO highly adaptable for facile surface modifications. Furthermore, the physicochemical and biological properties of GO can be enhanced through the integration with other bioactive materials. For example, the attachment of polyethylene glycol (PEG) onto GO yielded PEG‐encapsulated GO, which exhibited improved biocompatibility, enhanced water dispersion, reduced cytotoxicity, and adjustable binding affinity to drugs and biomolecules.^[^ ^84^ ^]^

**Figure 5:**
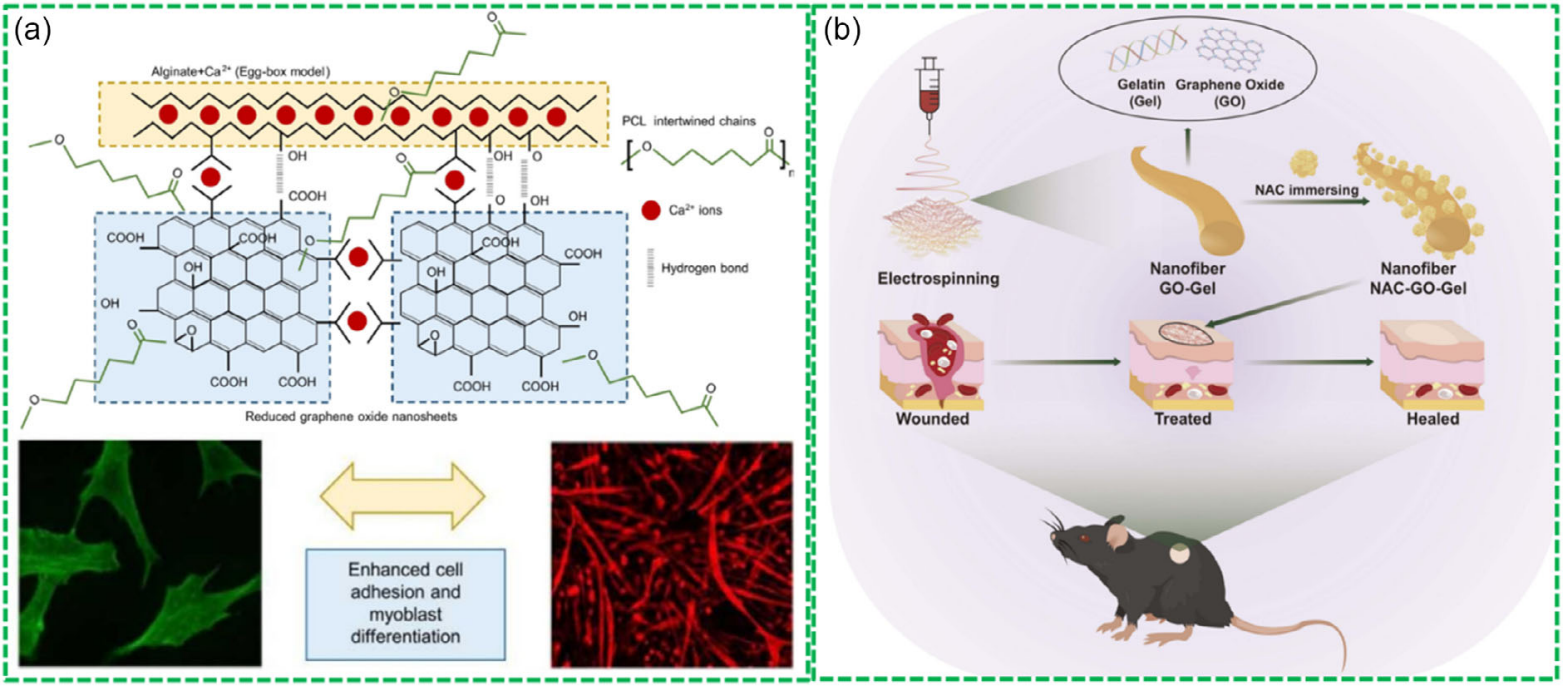
Bio‐RGMs with high biocompatibility for biomedicine: a) synthesis of CA/PCL/RGO hydrogels for cell adhesion and differentiation. Reproduced with permission.^[^ ^85^ ^]^ Copyright 2022, Elsevier. b) Biocompatible NAC‐GO‐Gel for wound repair. Reproduced with permission.^[^ ^86^ ^]^ Copyright 2023, Dovepress.

Bio‐RGMs with high biocompatibility and low cytotoxicity have shown great potential for biomedical applications. In a study by Aparicio‐Collado et al. electroactive calcium‐alginate (CA)/PCL/RGO nanohybrid hydrogels were developed (**Figure** **5**a), demonstrating excellent biocompatibility with C2C12 murine myoblasts and facilitating myoblast adhesion and myogenic differentiation.^[^ ^85^ ^]^ These results suggest the potential of these nanohybrid hydrogels for regenerating electroactive tissues such as skeletal muscles.

In another research by Yu et al. a GO‐gelatin (GO‐Gel) scaffold was cross‐linked with N‐acetyl cysteine (NAC) was fabricated using electrospinning for skin wound repair.^[^ ^86^ ^]^ The formed NAC‐GO‐Gel scaffold exhibited optimal biocompatibility, sustained release of NAC, facilitated re‐epithelialization, enhanced neovascularization, and accelerated scarless wound healing in mice (Figure 5b). Consequently, the NAC‐GO‐Gel leveraged the beneficial properties of graphene and its derivatives while effectively addressing their limitations, rendering them suitable for a broad array of biomedical applications.

In general, the modification of GMs with biomolecules to form Bio‐RGMs can increase the biocompatibility and decrease the cytotoxicity of GMs. However, with the degradation of coated biomolecules from the surface of GMs, potential increased cytotoxicity of Bio‐RGMs could affect their biomedical and tissue engineering applications. Therefore, long‐term biological evaluations should be carried out toward Bio‐RGMs when applying them for practical clinical applications.

### 4.2 Antibacterial Ability

Bio‐RGMs reveal intrinsic antimicrobial ability, positioning them as promising “green” agents capable of destroying bacteria without the risk of resistance.^[^ ^87^ ^]^ Research on antibacterial effects of GMs has surged, with studies showcasing their efficacy through diverse mechanisms including mechanical interaction with bacterial membranes, induction of oxidative stress, disruption of the membrane integrity, and interference with bacterial glycolysis processes.^[^ ^88^, ^89^ ^]^ Moreover, the functionalization of Bio‐RGMs with antimicrobial agents like metal nanoparticles, natural compounds, and antibiotics has proven to augment their overall effectiveness.^[^ ^90^ ^]^

Recently, popular antimicrobial agents were utilized for the customization of GO/RGO biofunctionalization encompass metal and MO nanoparticles,^[^ ^91^, ^92^ ^]^ natural compounds and derivatives (e.g., CS, alginate, Gel, etc.),^[^ ^93^, ^94^, ^95^ ^]^ synthetic polymers,^[^ ^96^, ^97^, ^98^ ^]^ as well as various antibiotics (e.g., β‐lactams, macrolides, quinolones, etc.).^[^ ^99^ ^]^

**Figure 6:**
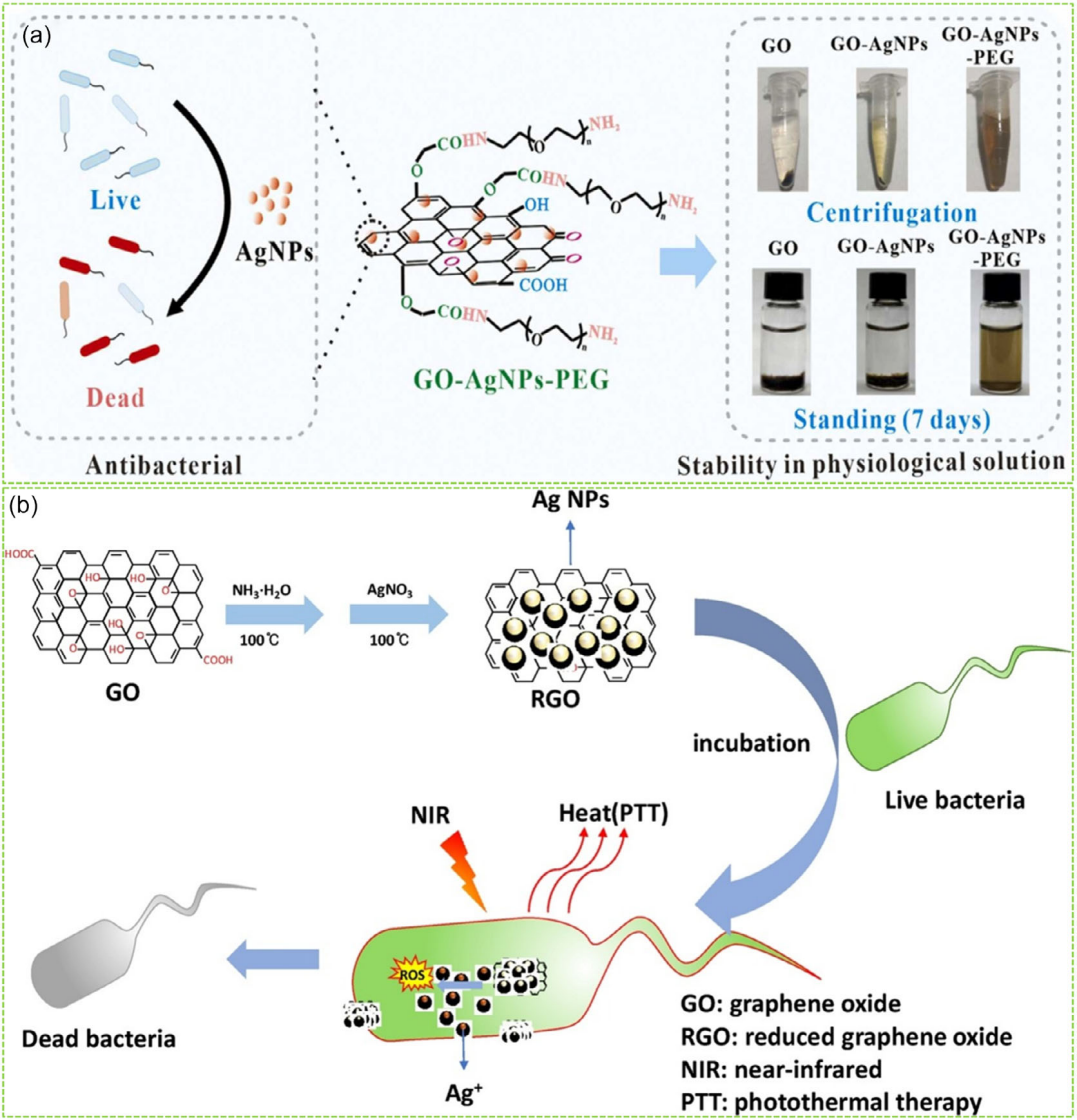
Bio‐RGMs with antibacterial ability: a) PEG‐GO‐AgNPs with high stability and antibacterial activity. Reproduced with permission.^[^ ^96^ ^]^ Copyright 2023, Elsevier. b) RGO/AgNPs nanocomposite with high antibacterial efficiency. Reproduced with permission.^[^ ^100^ ^]^ Copyright 2020, Elsevier.

In a recent study, Bao et al. reported the synthesis of PEG‐modified GO‐AgNPs nanocomposites for antibacterial application.^[^ ^96^ ^]^ As shown in **Figure** **6**a, gallic acid was used as a green reductant and stabilizer for the synthesis of stable GO‐AgNPs nanocomposites, which were then further functionalized with PEG to enhance their hydrophilicity in physiological solution. It was found that the formed PEG‐GO‐AgNPs nanocomposites exhibited high biocompatibility, stability, and antibacterial activity, due to the reinforcement of GO with both AgNPs and PEG.

In another study, Tan et al. developed RGO/AgNPs nanocomposites and assessed their antibacterial activity against *E. coli* and a multidrug‐resistant strain of *K. pneumoniae*. Their results revealed significantly higher antibacterial efficacy accompanied by increased reactive oxygen species (ROS) generation (Figure 6b).^[^ ^100^ ^]^ In a separate report on chitooligosaccharide‐modified GO (CG), researchers created a foam gel dressing composed of CG and a CA foam substrate.^[^ ^95^ ^]^ The study demonstrated that the formed CG/CA greatly enhanced hemostasis and endowed the dressing with excellent antibacterial activity, promoting the ability for wound healing. Furthermore, a recent investigation highlighted that GO‐Ag nanocomposites exhibited enhanced antimicrobial and biocompatible properties when functionalized with polyvinylpyrrolidone (PVP) molecules.^[^ ^97^ ^]^

Moreover, it is worth mentioning that these antimicrobial Bio‐RGMs often possess other unique biological functions in parallel, enabling them to disrupt pathogenic microorganisms and biofilm communities while synergistically promoting accelerated tissue repair. In a study by Baheiraei et al. RGO‐coated alginate (ALG) scaffolds (ALG‐RGO) were fabricated as novel electroactive cardiac patches.^[^ ^93^ ^]^ These patches exhibited excellent integrative properties, including enhanced mechanical strength and electrical conductivity, improved viability, and promoted adhesion of human umbilical vein endothelial cells. In addition, the patches boosted angiogenic capability and vascularization, alongside antibacterial activity against several pathogenic bacteria. In a similar study, Wu et al. reported the construction of GO/ZnO/HA composite microspheres, which achieved not only ideal inhibition of bacteria but also enhanced biocompatibility, osteogenic induction, and physicochemical properties.^[^ ^101^ ^]^

In summary, the development of antimicrobial functionalized Bio‐RGMs holds great potential for a wide range of applications in bioremediation and tissue repair, highlighting the versatility and effectiveness of these novel antibacterial 2D materials.

### 4.3 Drug Nanocarriers with Controlled and Targeted Release

**Figure 7:**
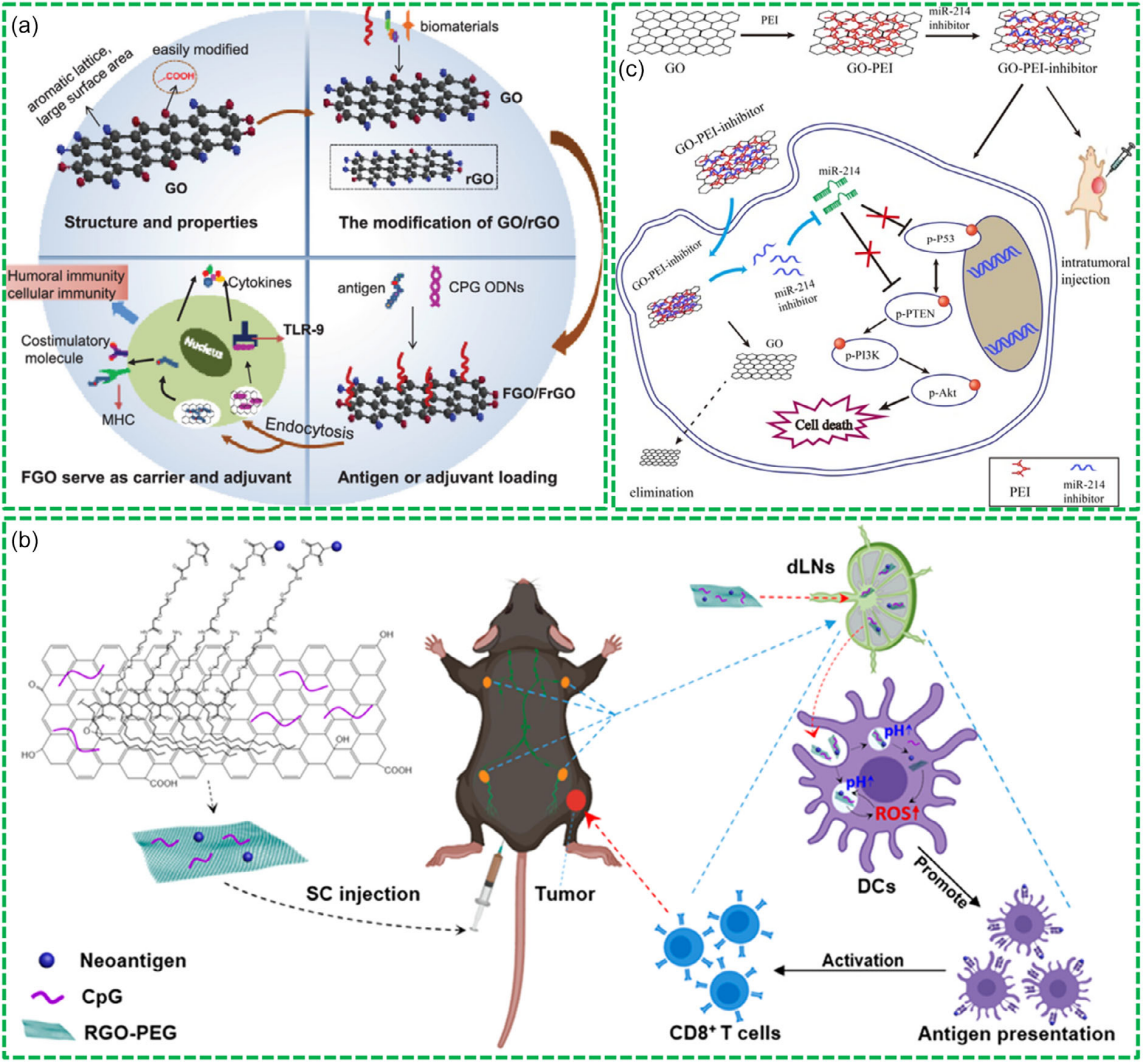
Drug nanocarrier function of Bio‐RGMs: a) biofunctionalized GO with superior properties as both carriers and adjuvants. Reproduced with permission.^[^ ^102^ ^]^ Copyright 2020, Elsevier. b) RGO‐PEG nanoplatform for cancer vaccination. Reproduced with permission.^[^ ^104^ ^]^ Copyright 2020, American Chemical Society. c) GO‐PEI as nanocarrier of miR‐214 inhibitors. Reproduced with permission.^[^ ^109^ ^]^ Copyright 2020, Dovepress.

Bio‐RGMs are highly valuable for their potential as biodegradable delivery vehicles with effective, controlled, and specific drug release capabilities. They can also serve as vaccine adjuvants, facilitating both humoral and cellular immunity (**Figure** **7**a).^[^ ^102^ ^]^ Their exceptional loading and targeting capacities for therapeutic agents, along with efficient cellular uptake, contribute significantly to their actions in drug delivery systems. The use of Bio‐RGMs with controlled and targeted release abilities helps to minimize the side effects and ensures the precise localization of drug delivery systems within specific cells, thereby enhancing overall therapeutic effectiveness.^[^ ^103^ ^]^

Bio‐RGMs exhibit promising potential in cancer chemotherapy and immunotherapy. Studies have reported that RGO‐PEG^[^ ^104^ ^]^ (Figure 7b) and PEI‐GO^[^ ^105^ ^]^ nanosheets demonstrated significant and sustained efficacy in inducing antigen‐specific T‐cell responses and inhibiting tumor growth and metastasis more effectively than traditional materials and approaches. Abdollahi et al. developed PEG bis‐amin (PEGA)‐decorated GO/magnetic nanoparticles (GOMNPs) for efficient drug loading and controlled release of methotrexate (MTX).^[^ ^106^ ^]^ The resulting GOMNP/PEGA/MTX composite demonstrated not only effective drug release dynamics but also enhanced killing efficiency against tumor cells, as well as good blood compatibility. Charmi et al. synthesized PEGylated GO (GO‐PEG) followed by the immobilization of the anticancer drug, Cur. The loading of Cur onto GO‐PEG exhibited higher release rate in basic environments compared to acidic conditions, offering potential benefits for cancer treatment, as human tumors tend to have slightly more alkaline pH levels than normal tissues.^[^ ^107^ ^]^

The use of Bio‐RGMs for gene delivery, crucial for efficient gene therapy, has garnered great attention in the last years. Alongside viral systems, nanoparticle‐based gene transfection systems are increasingly explored due to their lower immunogenicity and ease of mass production. A recent study demonstrated that a PEI‐coated GO (GO‐PEI) nanocarrier exhibited high gene transfection efficiency in HEK293T and Calu‐6 cell lines.^[^ ^108^ ^]^ In another investigation, GO‐PEI complexes efficiently delivered miR‐214 inhibitors (a microRNA associated with cell invasion and migration) to oral squamous cell carcinoma cells (Figure 7c), controlling their intracellular release, and significantly inhibiting tumor volume growth in xenograft mouse models.^[^ ^109^ ^]^ Liu et al. functionalized GQD and RGO with PEG and PEI to modulate laser‐induced photoporation, achieving enhanced intracellular delivery of extrinsic drugs or genes.^[^ ^110^ ^]^ The modified GQD and RGO with PEG or PEI demonstrated improved colloidal stability, leading to a more uniform photothermal effect, enhanced transfection efficiency, and cell viability. Notably, RGO could be excited by a broad range of laser wavelengths, including near‐infrared irradiation, potentially facilitating the transfection in thicker biological tissues.

Furthermore, Bio‐RGMs have emerged as promising candidates as novel vaccine adjuvants to enhance antigen immunogenicity and induce long‐lasting immunity. Studies have shown that Bio‐RGM nanocarriers can augment antigen presentation and enhance the function of immune cells through specific signaling pathways, thereby activating innate immune responses and inflammatory factors,^[^ ^111^ ^]^ attributed to their significant oxidative stress and inflammation. For instance, Huang et al. developed a novel Bio‐RGM as a drug nanocarrier for an influenza vaccine, comprising self‐assembled nanoparticles containing GQDs, carnosine, resiquimod, and Zn^2+^ ions.^[^ ^112^ ^]^ They demonstrated that the engineered Bio‐RGM enhanced antigen loading, improved dendritic cell recruitment, and activated antigen‐presenting cells, thereby facilitating both humoral and cellular immune responses to the influenza vaccine. In another study, Bai et al. utilized CS‐modified GO as a nanocarrier (GO/CS) to deliver the Hepatitis E virus antigen P239 protein (GO/CS/P239).^[^ ^113^ ^]^ The designed GO/CS/P239 exhibited good biosafety and elicited a robust immune response, resulting in improved production of IgG antibodies and cytokines compared to the vaccine containing only a single P239 protein fraction.

### 4.4 Capacity of Label‐Free Biosensors

The remarkable optical, electrical, and physical properties of Bio‐RGMs, combined with their facile modification, render them promising candidates for biosensing applications. Graphene‐based biosensors have attracted considerable attention owing to their high electron and thermal conductivity, outstanding sensitivity and specificity, rapid analysis, excellent biocompatibility, cost‐effectiveness, and potential for nanominiaturization.^[^ ^15^ ^]^

For instance, Kang et al. reported a biodetection system that utilizes transcriptional activator‐like effectors (TALEs) and GO to directly detect antibiotic resistance genes in a simple, rapid, and sensitive manner. This system relies on the prompt dissociation of TALEs from GO in the presence of target DNA sequences, eliminating the need for denaturation.^[^ ^83^ ^]^ Similarly, Xiang et al. demonstrated the effectiveness of a highly sensitive glassy carbon electrode modified with GQDs combined with specific sequence DNA molecules as probes for targeting hepatitis B virus DNA.^[^ ^114^ ^]^ In another study, the RGO‐PDA‐AuNP composite was developed as an electrochemical sensor capable of rapidly label‐free detecting Mycobacterium tuberculosis.^[^ ^115^ ^]^ These advancements hold significant potential for improving the early diagnosis of infections and diseases, as well as facilitating the development of targeted therapies and prognostication.

Bio‐RGMs have also demonstrated considerable potential as effective optical biosensors, particularly in the applications such as fluorescence resonance energy transfer (FRET), surface‐enhanced Raman spectroscopy, surface plasmon resonance, and colorimetric sensors.^[^ ^116^ ^]^ These capabilities stem from the unique optical properties of GMs, which include broadband and tunable absorption, fluorescence bursts, and strong polarization‐related effects. Although pristine graphene does not inherently exhibit photoluminescence properties, graphene derivatives such as GO, RGO, GQDs, and g‐C_3_N_4_ can be leveraged as donors or acceptors in FRET assays due to their optical characteristics. For instance, GO or RGO integrated with ssDNA could act as a fluorescence acceptor while simultaneously serving as aptamer adsorbates for probing living cells or biomolecules.^[^ ^117^, ^118^ ^]^ Furthermore, GO combined with fluorescent magnetic nanoparticles has been shown to function as a fluorescence quenching component and serve as a suitable FRET pair for DNA detection.^[^ ^119^ ^]^

The above sections introduce the properties and functions of some Bio‐RGMs. To make it more clear, here we present a **Table** **3** to summarize the interactions, structure, and function‐specific applications of Bio‐RGMs.

**Table 3:** Summary of interactions and structure of Bio‐RGMs toward function‐specific applications.

GMs	Biomolecules	Interactions	Structure	Application	References
RGO	PCL/CA	Coordination	Hydrogels	Muscle tissue engineering	[85]
GO	Gelatin/NAC	Covalent binding	Scaffold	Wound repair	[86]
RGO	ALG	Coordination	Scaffold	Cardiac patch	[93]
GO	PEG	Covalent binding	Nanocomposites	Antibacteria	[96]
RGO	PEG	Covalent binding	Nanohybrids	Drug nanocarrier	[104]
GO	PEI	Covalent binding	Hydrogels	Drug nanocarrier	[105]
GO	PEGA	Covalent binding	Nanocomposites	Drug delivery	[106]
GO	PEG	Covalent binding	Nanosheets	Drug delivery	[107]
GO	PEI	Covalent binding	Hybrids	Nanocarriers of cells	[108]
GO	PEI	Covalent binding	Hybrids	Drug delivery	[109]
GO/QQD	PEG/PEI	Covalent binding	Hybrids	Drug delivery	[110]
GO	CS	Covalent binding	Hybrids	Drug nanocarrier	[113]
GQDs	DNA	Covalent binding	Nanocomposites	EC biosensors	[114]
RGO	PDA	Covalent binding	Hybrids	EC biosensors	[115]

## 5 BTE Applications of Bio‐RGMs

The skeletal system possesses a certain degree of self‐repair and regenerative capability. However, significant bone defects resulting from conditions such as osteoporosis, tumors, traumatic fractures, and infections have been not regenerated spontaneously.^[^ ^120^ ^]^ These severe bone defects affect millions of people worldwide each year, leading to serious disabilities. Autologous and allogeneic bone grafting are the gold standards for treating bone defects. However, they are limited by the issues such as donor site morbidity, high failure rates (up to 50%), and limited availability of graft materials. Therefore, it is necessary to develop artificial bone graft substitutes to meet the significant clinical demands.

The ideal bone graft substitute should possess good biocompatibility, osteoinductivity, osteoconductivity, osteointegration, and mechanical properties that suitable for bone tissue matching. BTE has introduced alternatives in both structure and function, known as biomimetic scaffolds. The regeneration of bone tissue mediated by biomimetic scaffolds represents a promising approach for bone repair and replacement. The primary issue with currently available artificial bone substitutes (scaffolds) is that they generally serve as fillers that lack osteoinductive capability. Bio‐RGMs have gained wide application in BTE fields due to their excellent mechanical properties and high biocompatibility that induced by both biomolecules and GMs.

### 5.1 Bone Repair and Regeneration

Bio‐RGMs can provide structural support for the growth and regeneration of bone‐related cells and tissues. Scaffolds containing Bio‐RGMs exhibited good biocompatibility and biomimicry, promoting the attachment, proliferation, and differentiation of bone‐related cells, thus accelerating the repair and regeneration of bone tissues.

Previous reports have indicated that GMs (such as GO and RGO nanosheets) could act as standalone promoter of bone repair and regeneration, due to their potential degradation, mechanical stability, and high surface area in aqueous solutions. For instance, Newby et al. observed that human mesenchymal stem cells (hMSCs) could spontaneously undergo osteogenic differentiation in the presence of GO nanoparticles, suggesting that the physicochemical properties of GO nanoparticles may trigger the expression of bone‐specific extracellular matrix (ECM) proteins.^[^ ^24^ ^]^ Therefore, the in‐depth understanding the interactions between cells and GMs and their effects on osteogenic differentiation will promote the application of GMs in bone repair and regeneration. Similarly, Park et al. utilized GO nanosheets with a narrow size distribution to induce osteogenic differentiation of bone marrow‐derived hMSCs (BM‐hMSCs) and observed improved osteogenesis in the experimental group treated with GO,^[^ ^121^ ^]^ which suggested that size‐controllable GO nanosheets may effectively promote the osteogenesis in BM‐hMSCs. These studies demonstrate the potential of GMs to influence the expression patterns of specific ECM proteins during the adhesion and osteogenic differentiation of hMSCs.

The formation of Bio‐RGMs, achieved by binding biomolecules onto GMs through both covalent and noncovalent interactions, enhances the functions and properties of GMs for BTE applications. This enhancement further supported the cell adhesion, proliferation, and stem cell differentiation.^[^ ^122^ ^]^ An increasing number of studies are exploring the combined use of GMs with bioactive nanomaterials such as Gel,^[^ ^123^ ^]^ silk fibroin methacrylate (SerMA),^[^ ^124^ ^]^ CS,^[^ ^125^ ^]^ PDA,^[^ ^126^ ^]^ PLGA nanofibers,^[^ ^127^ ^]^ HA,^[^ ^30^, ^128^ ^]^ ALG,^[^ ^129^ ^]^ proteins (such as silk fibroin),^[^ ^130^ ^]^ and peptides^[^ ^131^ ^]^ to enhance the cell adhesion, proliferation, osteogenic differentiation, and mineralization of bone‐related cells, thereby promoting bone repair and regeneration.

**Figure 8:**
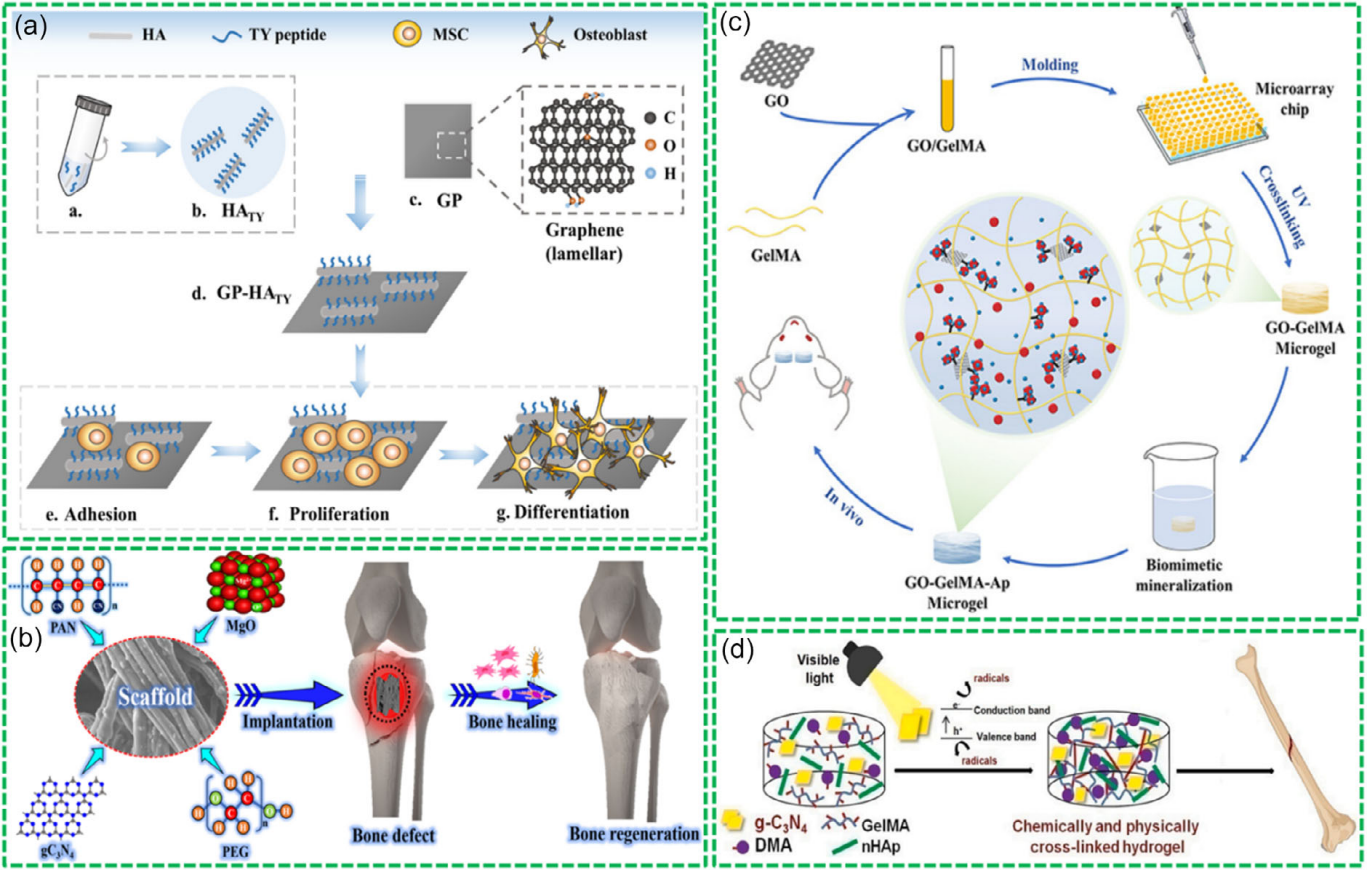
Bio‐RGMs for bone repair and regeneration: a) GP‐HA‐TY composites for BMSC differentiation. Reproduced with permission.^[^ ^30^ ^]^ Copyright 2021, American Chemical Society. b) PAN/PEG‐MgO/g‐C_3_N_4_ membrane for bone regeneration. Reproduced with permission.^[^ ^132^ ^]^ Copyright 2024, American Chemical Society. c) GO‐GelMA‐Ap microgels for bone repair. Reproduced with permission.^[^ ^134^ ^]^ Copyright 2023, Dove Medical Press. d) GelMA*‐co*‐DMA/nHAP/g‐C_3_N_4_ composite hydrogel for bone regeneration. Reproduced with permission.^[^ ^135^ ^]^ Copyright 2024, Elsevier.

Various Bio‐RGM‐based 2D films and membranes have been utilized for mediating bone repair and regeneration. For instance, Wang et al. utilized GP combined with a graphene‐binding peptide (TWWNPRLVYFDY) as an affinity linker and HA nanorods to achieve the Bio‐RGMs,^[^ ^30^ ^]^ as shown in **Figure** **8**a. The designed peptide as a bifunctional linker, bridges the formation of GP‐peptide‐HA complexes with high hydrophilicity. The peptide bound to GP could mediate the biomimetic synthesis of bioactive inorganic nanoparticles (such as HA), achieving flexible biological functionalization of GP with various inorganic NPs while maintaining the integrity of the carbon lattice of GP. Therefore, the designed Bio‐RGMs with bioactive peptides and HA‐induced in vitro osteogenic differentiation of rat bone marrow mesenchymal stem cells (BMSCs). Li et al. prepared a pearl‐like GO/elastin binary nanocomposite membrane through a simple evaporation method.^[^ ^120^ ^]^ The incorporation of 20% elastin with GO increased Young's modulus and tensile strength of the composite membrane, equivalent to cortical bone. When used for inducing osteogenic differentiation of BMSCs, a significant increase in osteogenic cell differentiation was observed.

To mediate bone repair and regeneration, appropriate substrates are required for the cell adhesion and growth. In addition, the adhered cells should have enough bioactivity and growth behavior. Danagody et al. reported electrospun synthesis of polyacrylonitrile (PAN)/PEG membrane doped with MgO/g‐C_3_N_4_ nanocomposites for enhanced bone regeneration.^[^ ^132^ ^]^ As depicted in Figure 8b, the introduction of MgO/g‐C_3_N_4_ nanocomposites into PAN/PEG nanofibers increased the pore density and reduced the hydrophobicity of PAN, demonstrating beneficial effects to the activity and growth of THP‐1 cells. After the implantation of the created Bio‐RGMs into the sites of bone defect, the Mg^2+^ ions could be released and promoted osteoblast differentiation and proliferation. In addition, both MgO and g‐C_3_N_4_ could enhance material capability to penetrate into the impaired tissues, and promote bone healing rate.

Pure biomaterials, such as COL and CS substrates, showed shortcomings like poor rigidity, low stability, and limited osteogenic properties. To improve the properties an functions of COL biomaterials for BTE, Davaie et al. synthesized a PDA/GO‐reinforced collagen membrane to enhance the properties of COL materials.^[^ ^126^ ^]^ It was found that the modified PDA/GO‐COL 2D membrane improved the mechanical properties of original collagen membrane and enhanced the proliferation and adhesion of HBMSCs, significantly improving their osteogenic performance. In addition, the reinforcement of PDA/GO coating improved the tensile strength and biodegradability of pure COL membranes, promoting their applications in BTE field. Puah et al. prepared a novel peptide‐conjugated multilayer GO (peptide/*m*‐GO) membrane for culturing human Wharton's jelly‐derived mesenchymal stem cells (WJ‐MSCs).^[^ ^133^ ^]^ The simple preparation process of the peptide/*m*‐GO membranes and their good biocompatibility played a significant role in controlling the osteogenic lineage of WJ‐MSCs, making it suitable for bone tissue regeneration.

In addition, various hydrogels that are based on Bio‐RGMs have also been utilized for BTE applications. Peng et al. successfully constructed porous GO‐GelMA microgels through a chemical cross‐linking reaction, obtaining Bio‐RGM microgels after the oxygenation of GO.^[^ ^134^ ^]^ As shown in Figure 8c, the formed GO‐GelMA microgels were immersed into a simulated body fluids solution to induce the formation of apatite (Ap) via biomimetic mineralization, synthesizing multifunctional GO‐GelMA‐Ap microgels. The abundant —COOH and —OH groups on GO are useful for providing active sites for biomimetic mineralization and mediating the proliferation of BMSCs. Systematic in vitro and in vivo experiments demonstrated that the constructed microgels exhibited good physicochemical properties, and the cultured BMSCs revealed higher osteoinductive ability and biomineralization efficiency, promoting cell proliferation and the repair capacity of rat bone defects.

Photo‐induced bioactive hydrogels showed wide applications in BTE and biomedical fields. Jiao et al. demonstrated the synthesis of a multifunctional Bio‐RGM for the adhesion and growth of BMSCs.^[^ ^123^ ^]^ RGO nanosheets were First modified with Gel to form Gel‐RGO (GOG), which was then combined with the photo‐crosslinked Gel hydrogel for mediating the differentiation of BMSCs. It was found that the designed Gel‐RGO hydrogel‐induced BMSCs to undergo dual differentiation into osteogenesis and vasculogenesis, promoting the osteogenic effect of biomimetic original callus. In a similar study, Qi et al. designed and fabricated photo‐crosslinked SerMA/GO hydrogel (SMH/GO) as a biomimetic scaffold for functional bone repair, capable of inducing BMSC proliferation, migration, and osteogenic differentiation.^[^ ^124^ ^]^ The formed SMH/GO hydrogel scaffold exhibited good biocompatibility, cell adhesion, proliferation‐promoting, and directionally migratory effects, as well as osteoinductive properties, accelerating bone repair. This study developed a type of novel bone substitute material by using sericin and GO, which can meet clinical requirements and overcome the limitations of artificial bones of lacking osteo‐induction abilities. Currently, Papaioannou et al. reported the construction of bioactive and biomimetic 3D hydrogel scaffolds for BTE application.^[^ ^135^ ^]^ As shown in Figure 8d, under the irradiation of visible light, g‐C_3_N_4_‐induced chemical and physical cross‐linking of GelMA, nHAp, and dopamine methacrylamide (DMA), forming multifunctional GelMA*‐co*‐DMA/nHAp/g‐C_3_N_4_ composite hydrogel. Due to the using of g‐C_3_N_4_ as a novel sustainable photoinitiator, the formation of the composite hydrogel is green, quick, and cost‐effective. The created composite hydrogel had similar bone composition by adjusting the content of nHAp and strong mechanical stability as bone growth support. In addition, the adhered cells revealed high cell viability and improved osteoblast differentiation.

Besides, Bio‐RGMs can promote the bone repair and regeneration through inducing the osteogenesis of osteoblasts. For instance, Liu et al. prepared a GO/CS/HA composite membrane using a one‐step vacuum filtration and biomimetic mineralization method for bone repair application.^[^ ^60^ ^]^ The composite membrane, with a multilayer structure, was enhanced in mechanical strength and surface hydrophilicity by CS and HA. This enhancement facilitated the adhesion, proliferation, differentiation, and mineralization of osteoblasts, promoting the bone regeneration. In another case, Sadek et al. investigated the stimulation of critical‐sized bone defect regeneration by g‐C_3_N_4_ and GO‐based biomaterials through in vitro and in vivo experiments.^[^ ^136^ ^]^ It was found that the g‐C_3_N_4_ and GO‐based Bio‐RGMs exhibited good biocompatibility with cells and blood, inducing osteogenesis of human fetal osteoblasts in vitro and promoting bone healing in vivo.

### 5.2 Bio‐RGMs for Bone‐Graft Alternatives

3D Bio‐RGM scaffolds constructed using GMs and active biomolecules exhibit excellent mechanical properties, structural stability, and enhanced biocompatibility and bioactivity. They can serve as artificial bone‐graft substitutes for repairing severe bone fractures, defects, or diseases. Ideal BTE scaffolds should have appropriate pore size, surface area‐to‐volume ratio, mechanical strength, biocompatibility, surface reactivity, and clinical applicability in terms of shape, all of which contribute to the promotion of cell adhesion, as well as vascular and neural growth.

**Figure 9:**
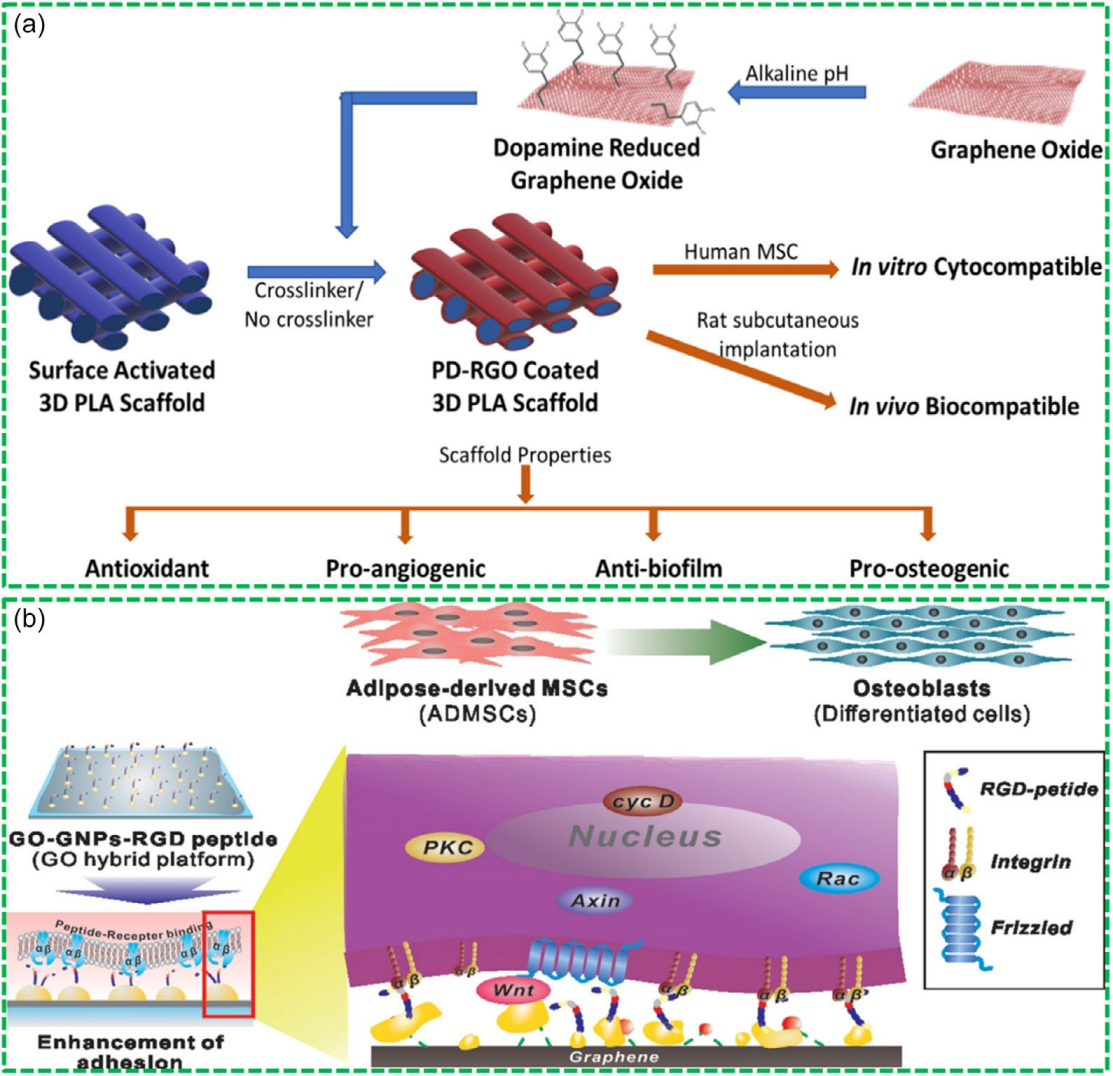
Bio‐RGM scaffolds for bone‐graft alternatives: a) 3D‐printed DA‐RGO‐reinforced PLA scaffold for BTE. Reproduced with permission.^[^ ^138^ ^]^ Copyright 2021, Elsevier. b) GO‐GNPs‐RGD scaffold for osteogenesis of hAMSCs. Reproduced with permission.^[^ ^131^ ^]^ Copyright 2021, Elsevier.

As an integral aspect of additive manufacturing, 3D printing has gained significant attention for producing bioactive implant 3D scaffolds for biomedicine and tissue engineering applications. In the field of regenerative medicine, 3D‐printed BTE scaffolds hold immense promise for applications in stem cell therapy aimed at regenerating bone defects. GO has emerged as a valuable bioink additive for fabricating Bio‐RGM‐based cell scaffolds via 3D printing. When incorporated into ALG‐based bioinks, GO enhanced the mechanical strength of bioink scaffolds while supporting the viability of mesenchymal stem cells.^[^ ^137^ ^]^ For example, Sharma et al. engineered a 3D‐printed polylactic acid (PLA) scaffold doped with DA‐RGO for BTE application.^[^ ^138^ ^]^ The DA‐RGO scaffold exhibited a multifaceted functionality, including antioxidant, antibiofilm, pro‐angiogenic, and osteogenic properties, as illustrated in **Figure** **9**a. By addressing challenges such as biofilm formation and oxidative stress induced by ROS, the scaffold promoted the integration with host tissues and supported the bone formation. Moreover, the scaffold‐induced hMSCs demonstrated enhanced antioxidant and pro‐angiogenic capabilities, along with osteogenic potential, further enhancing the therapeutic efficacy of the designed Bio‐RGM scaffolds.

The CVD method is highly useful and facile for the construction of GM‐based scaffolds for BTE. Kang et al. electrochemically modified indium tin oxide (ITO) coatings with CVD‐formed graphene and gold nanoparticles (GNPs), and developed a hybrid scaffold based on GO, termed GOHP, to guide and accelerate osteogenesis of hAMSCs.^[^ ^131^ ^]^ The fabrication process is shown in Figure 9b, in which the scaffold is further modified with cysteine‐modified RGD peptide through the formation of Au—S bonds. Therefore, the RGD‐reinforced GOHP (GO‐GNPs‐RGD) promoted the differentiation of hAMSCs into osteoblasts or progenitor stem cells, showing the potential of the Bio‐RGMs for treating various diseases/disorders, including osteoporosis, rickets, and osteogenesis imperfecta.

Electrospinning is also a useful technique for the construction of bio‐scaffolds for BTE as the electrospun scaffolds can provide porous structure, interconnectivity, high surface area, and high volume. Incorporating GO into scaffolds, along with electrospun peptides, enhanced their thermal, physicochemical, and mechanical properties, while promoting stem cell proliferation in vitro.^[^ ^139^ ^]^ Motiee et al. developed a GO‐enhanced scaffold using poly(3‐hydroxybutyrate)‐CS (PC) via electrospinning. This modification significantly improved the mechanical properties of the formed scaffold, reduced its biodegradation rate, and enhanced the biomineralization of Bio‐RGM scaffolds.^[^ ^140^ ^]^ Furthermore, the PC/GO (PCG) nanocomposite scaffold increased the adhesion, migration, proliferation, and alkaline phosphatase (ALP) activity of MG‐63 osteosarcoma cells cultured on it. Ghorbani et al. fabricated PLA‐coated PU‐GO scaffolds using electrospinning.^[^ ^61^ ^]^ These scaffolds, immersed in a DA solution and coated with PDA, exhibited synergistic effects on bioactivity and osteogenic expression. The physicochemical and in vitro properties of the composite nanofibrous scaffolds, enhanced by the presence of PDA coating, surpassed those of conventional scaffolds. The results demonstrated the scaffold's suitability for both in vivo and in vitro bone regeneration. In a similar study, Aidun et al. utilized electrospinning to create PCL/CS/COL scaffolds mixed with GO.^[^ ^141^ ^]^ These scaffolds exhibited osteogenic properties, contributed to enhanced BTE, and displayed favorable morphology, biocompatibility, and biological activity.

Besides, chemical cross‐linking combined with freeze‐drying and self‐assembly can also promote the formation of various Bio‐RGM scaffolds for BTE applications. Bahrami et al. synthesized RGO‐coated COL (COL‐RGO) scaffolds through chemical cross‐linking and freeze‐drying methods.^[^ ^142^ ^]^ The formed COL‐RGO scaffold, characterized by 3D porosity, exhibited 2.8 times greater mechanical strength than the COL scaffold, along with enhanced adhesion and expansion properties, leading to increased vitality and proliferation of hBMSCs. Upon implantation in a rabbit skull defect model, the COL‐RGO scaffold facilitated the bone formation. Similarly, Liu et al. fabricated GO‐COL aerogels using chemical cross‐linking and lyophilization techniques.^[^ ^143^ ^]^ The constructed Bio‐RGM (GO‐COL) scaffolds demonstrated excellent biocompatibility, high porosity, and hydrophilicity, making them suitable for bone regeneration applications. Experimental findings revealed that the GO‐COL aerogel exhibited superior biomineralization rate and cytocompatibility compared to other aerogel groups. Furthermore, in a rat skull defect model, the GO‐COL aerogel demonstrated enhanced bone repair efficacy compared to pure COL aerogel.

Rebecca et al. utilized electrostatic self‐assembly technique to cross‐link type I COL with G, producing a 3D G‐COL composite scaffold.^[^ ^144^ ^]^ The scaffold exhibited enhanced mechanical strength, electrical conductivity, and a 3D porous structure, showing great effects on promoting bone healing. Similarly, Mahanta et al. developed a novel CS nanohybrid scaffold incorporating GO and functionalized GO as nanofillers.^[^ ^145^ ^]^ This scaffold featured an open interconnected porous network structure, improved hydrophilicity, enhanced mechanical strength, and demonstrated superior biocompatibility, facilitating accelerated bone growth.

### 5.3 Drug Delivery of Bio‐RGMs for BTE

Bio‐RGMs can be used as excellent nanocarriers of drug delivery systems to effectively deliver growth factors, drugs, and other bioactive substances to bone defect sites, to promote bone cell proliferation and bone tissue regeneration.

First, the designed Bio‐RGMs can serve as nanocarriers for loading bone morphogenetic protein‐2 (BMP‐2) to promote the bone repair and regeneration. Wu et al. reported the bone regeneration using a electrospinning SF scaffold by grafting BMP‐2 peptide‐functionalized GO.^[^ ^146^ ^]^ The BMP‐2 peptide P24 was grafted onto GO, and the functionalized GO was coated onto the electrospun silk fibroin (SF) scaffold through electrostatic interaction. The composite scaffold exhibited good biocompatibility, and the hydrophilicity, cell adhesion, and proliferation of the coated scaffold were improved compared to uncoated scaffold. After the fixation of GO‐P24, the scaffold showed excellent biological activity, promoted the bone regeneration of critical‐sized bone defects, and enhanced osteogenic differentiation of BMSCs. Xu et al. introduced amino, carboxyl, and hydroxyl chemical groups on graphene to covalently bind to the peptide that derived from BMP‐2 and compound with PLGA to prepare 3D Bio‐RGM scaffolds.^[^ ^147^ ^]^ The in vivo experiments of this scaffold confirmed that it promoted heterotopic bone regeneration in rats and repaired skull defects in rabbits. Zhang et al. prepared a bioactive PLGA/β‐tricalcium phosphate (β‐TCP) composite scaffold using the original low‐temperature 3D printing and loaded GO and BMP‐2‐like peptide (PTG/P) in situ.^[^ ^127^ ^]^ The formed PTG/P scaffold promoted the growth of rat BMSCs into the scaffold, mediated osteogenic differentiation, and significantly facilitated bone regeneration in severe bone defects.

In addition, as bone‐forming peptides (BFP) can promote the bone growth and regeneration, the BFP‐reinforced GMs are useful in BTE. In a typical study, Wang et al. constructed a GO‐based nanofiber scaffold. The scaffold contains a BFP‐1 modified by GO, which could promote and induce the proliferation and osteogenic differentiation of human pluripotent stem cells (ihPSC).^[^ ^148^ ^]^ It has great practical value in the transformative potential of BTE.

**Figure 10:**
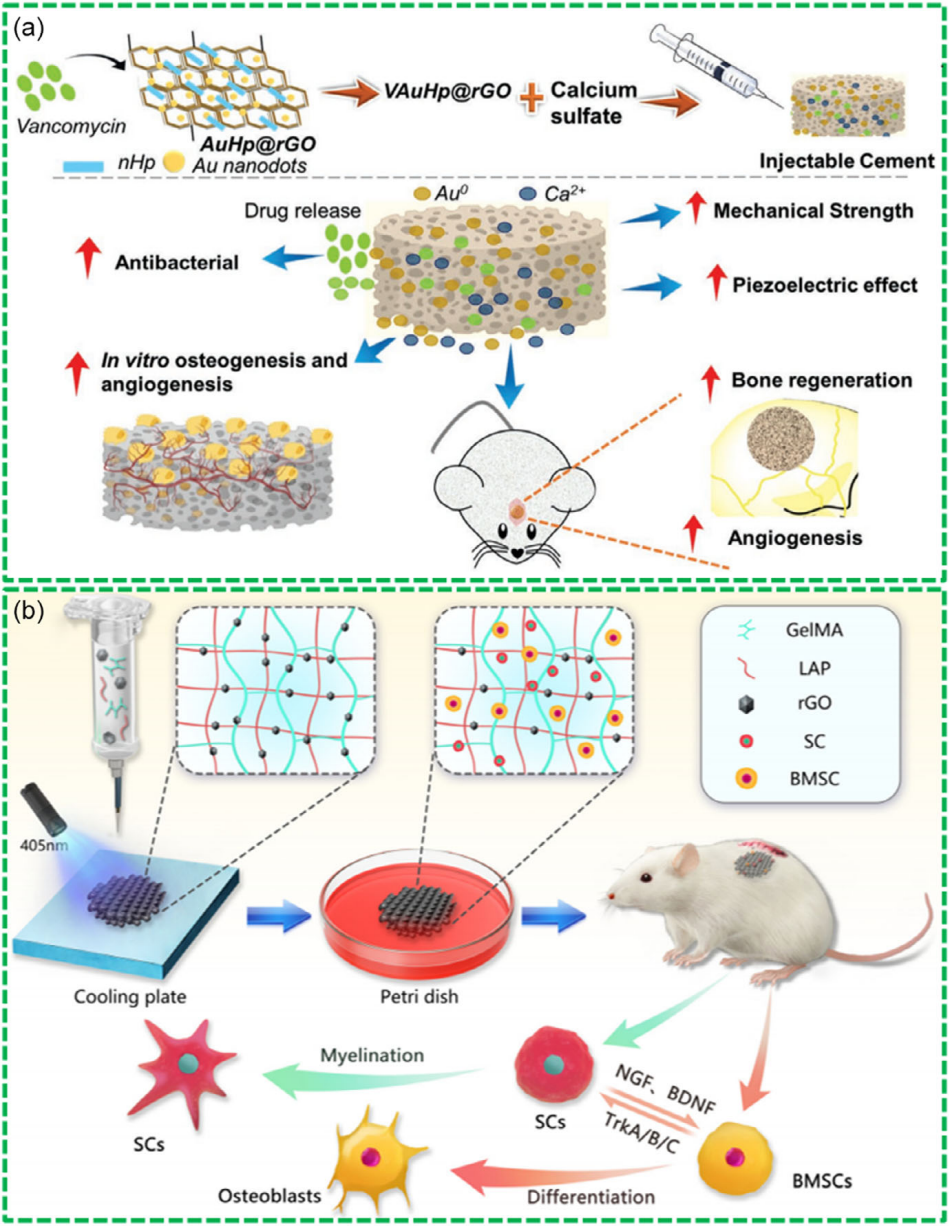
Bio‐RGMs as drug nanocarriers for BTE: a) VAuHp@RGO‐reinforced injectable calcium sulfate cement for antibacterial bone repair. Reproduced with permission.^[^ ^149^ ^]^ Copyright 2023, Wiley‐VCH. b) 3D‐bioprinted RGO hydrogel‐loaded SCs and BMSCs for bone repair. Reproduced with permission.^[^ ^72^ ^]^ Copyright 2023, Royal Society of Chemistry.

In the process of bone repair, bone infection is a serious problem that affects the bone repair and regeneration efficiency of materials. To address this issue, Chopra et al. developed an injectable bone cement doped with antibacterial drug for bone regeneration application.^[^ ^149^ ^]^ As shown in **Figure** **10**a, RGO nanosheets were First decorated with gold nanodots (Au) and nanohydroxyapatite (nHp) to form AuHp@RGO composites, which further provided the support for the adsorption of a commonly used antibiotic, Vancomycin (V). Then, the formed VAuHp@RGO composites were mixed with calcium sulfate cement to form a multifunctional injectable cement. The modification of calcium sulfate cement with VAuHp@RGO composites reinforced material mechanical, antimicrobial, osteoinductive, and osteoconductive properties, making the injectable cement useful for clinical bone repair without bacterial infections.

Growth factors are effective drugs that can promote the growth of bone‐related cells, and play important roles in BTE applications of biomaterials. In a recent study, Wang et al. developed a core‐shell Bio‐RGM scaffold for accelerated bone defect repairment, using insulin‐like growth factor (IGF)‐bound phosphorus‐doped g‐C_3_N_4_ as core, and nerve growth factor (NGF)‐modified SF as shell via electrospinning.^[^ ^150^ ^]^ Due to the unique photoelectronic properties of g‐C_3_N_4_, red light could covert light and electricity in g‐C_3_N_4_ to improve the efficiency of osteogenesis. Therefore, under the irradiation of red light, the formed IGF@C_3_N_4_/NGF@SF scaffold promoted BMSC osteogenesis and enhanced BMSC‐induced neural differentiation cells into neuron. This study provides a promising example for using light regulation of material properties for improved BTE application.

Furthermore, another potential strategy for Bio‐RGMs as nanocarriers for bone repair is loading the bone‐forming cells into the biological system. For instance, Zhang et al. utilized a 3D‐bioprinted GO/ALG/GEL composite biological scaffold material to simulate the treatment of skull defects in mice.^[^ ^129^ ^]^ They found that GO composite bioinks improved bioprinting, increased scaffold fidelity, enhanced cell proliferation, and promoted osteogenic differentiation of hMSCs, along with increased ECM mineralization in BTE. In another similar study, the design and application of RGO involved in cell delivery were reported.^[^ ^72^ ^]^ At present, it is hard to achieve synchronized nerve and bone regeneration with single‐functional biomaterials. Therefore, the researchers utilized 3D printing technology to construct RGO/GelMA hydrogels, which were further used for the loading of Schwann cells (SCs) and BMSCs, as shown in Figure 10b. The created multifunctional Bio‐RGM hydrogels loaded cells revealed not only enhanced neuronal differentiation of SCs, but also improved adhesion and osteogenic differentiation of BMSCs. This study provides a promising strategy for the creation of Bio‐RGM‐based biomimetic biomaterials for promoting synergistic regeneration of nerves and bone, showing useful inspirations for clinical application.

Purohit et al. developed Gel, ALG, and GO nanocomposite scaffolds.^[^ ^151^ ^]^ The scaffold exhibited good swelling, porosity, biocompatibility, biodegradability, and mechanical strength. When MG‐63 osteosarcoma cells were inoculated on the nanocomposite scaffolds, enhanced cell attachment and proliferation were observed compared to GA scaffolds. The studies on cell differentiation via scaffold implantation of mesenchymal stem cells showed higher expression of osteoblast transcription factors (Runx2 and Osteocalcin) and higher ALP activity. The results indicated that the scaffold was suitable for BTE.

### 5.4 Bone Tumor Inhibition

Bio‐RGMs exhibit the ability to inhibit the growth of tumor cells and can serve as carriers for tumor‐killing drugs, thereby killing tumor cells, inhibiting tumor growth, and promoting osteogenic tissue regeneration.

**Figure 11:**
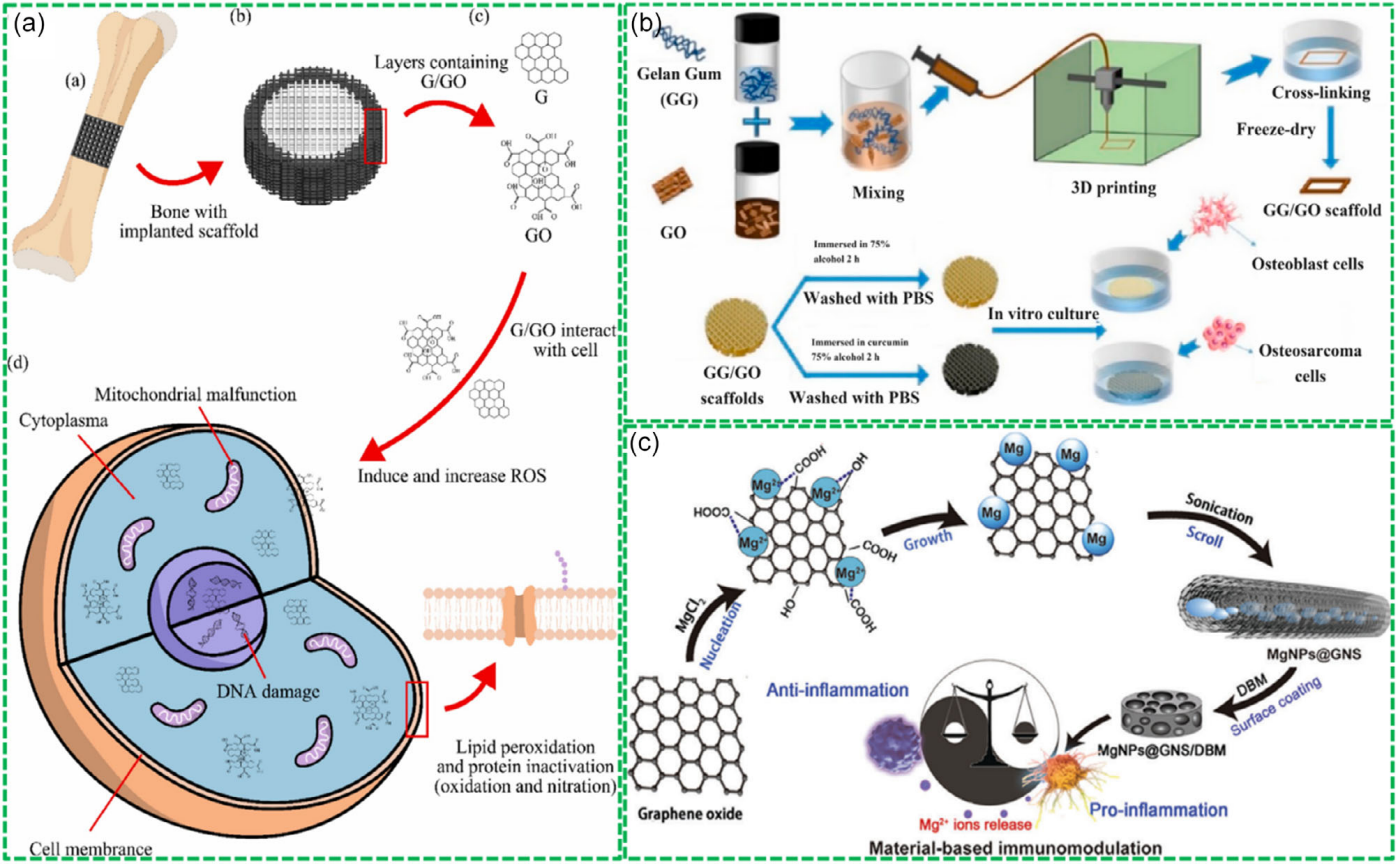
a,b) Bio‐RGMs for inhibiting bone tumors and c) adjusting microenvironment: (a) 3D G/GO/PCL scaffolds for treating bone tumor. Reproduced with permission.^[^ ^152^ ^]^ Copyright 2023, Elsevier. b) GG/GO/Cur scaffolds for treating bone tumor. Reproduced with permission.^[^ ^153^ ^]^ Copyright 2021, Elsevier. (c) MgNPs@GNSs for adjusting the microenvironment for bone regeneration. Reproduced with permission.^[^ ^154^ ^]^ Copyright 2020, Wiley‐VCH.

Hou et al. utilized 3D‐printed PCL scaffolds with varying concentrations of raw graphene (G) and GO for the bone cancer treatment and subsequent bone regeneration.^[^ ^152^ ^]^ As depicted in **Figure** **11**a, a dual‐functional 3D‐printed PCL scaffold was able to induce the attachment, proliferation, and differentiation of hADSC and Saos‐2 cells. It also facilitated the release of ROS, leading to mitochondrial dysfunction, lipid peroxidation, and inactivation of intracellular proteins. These effects ultimately resulted in apoptosis or necrosis of bone cancer cells, thereby inhibiting cancer cell growth while promoting bone tissue regeneration.

In a similar study that focused on the use of GO for bone tumor treatment, Zhu et al. developed a hydrogel bio‐ink with favorable thixotropic and restorative properties, employing gellan gum (GG) and GO as key materials. By incorporating Cur into the bio‐ink, they fabricated functional bone repair scaffolds (GG/GO/Cur) using 3D printing technology for tumor treatment and bone reconstruction.^[^ ^153^ ^]^ As illustrated in Figure 11b, the pH‐responsive GG/GO/Cur scaffold effectively targeted tumor cells and suppressed tumor growth by releasing Cur within the acidic microenvironment characteristic of tumor cells in vitro. Concurrently, the functional bone repair scaffolds facilitated the attachment and proliferation of osteoblasts. The 3D‐printed GG/GO/Cur scaffold maintained high fidelity with the model owing to its regular and interconnected porous structure. It significantly impeded the growth of the human osteosarcoma cell line MG‐63, induced tumor cell death effectively in vitro, and supported the attachment and proliferation of mouse osteoblasts (MC3T3). Functional GG/GO/Cur scaffolds demonstrated promising efficacy in repairing bone defects resulting from surgery, eliminating residual tumor cells, and achieving dual purpose of tumor treatment and bone reconstruction.

Bio‐RGMs possess the capability to modulate the inflammatory responses of macrophages M1 and M2, thereby creating a conducive microenvironment that can promote the bone defect regeneration. For instance, Zheng et al. engineered magnesium nanoparticles (MgNPs) in conjunction with GO nanosheets (MgNPs@GNSs) to regulate the immune microenvironment.^[^ ^154^ ^]^ As depicted in Figure 11c, the MgNPs@GNS nanoplatform orchestrates the M1 and M2 inflammatory responses of macrophages in a coordinated manner. Through the sequential inflammatory response elicited *via* chemotaxis, mitosis, and morphogenesis, the fabricated Bio‐RGM nanoplatform stimulated angiogenesis and osteogenesis in vitro. Furthermore, it could deposit acellular bone matrix scaffolds, leading to significant vascularization and enhanced bone regeneration in rat skull defect models. By leveraging the MgNPs@GNS nanoplatform, the synergistic immunomodulatory properties of GO (“Yang”) and magnesium (“Yin”) interact synergistically to establish a microenvironment conducive to healing, thereby facilitating acellular matrix‐mediated bone regeneration.

Sun et al. also engineered a scaffold capable of immune modulation of biological microenvironment and promoting the bone regeneration.^[^ ^130^ ^]^ The formed SF/nHA scaffold was fabricated via freeze‐drying with EDC/NHS reaction, and cross‐linked with GO. The in vitro experiments revealed that the SF/nHA scaffolds‐induced macrophage differentiation toward the M2 phenotype while inhibiting differentiation toward the M1 phenotype. In a rat skull defect repair experiment, it was observed that the scaffold containing 0.1% GO facilitated M2 differentiation of macrophages during the middle and late stages of biomaterial‐induced inflammation, nearly bridging the injured site 12 weeks postsurgery.

### 5.5 Bio‐RGMs for Preclinical BTE Applications

To make it more clear for the above introduction and discussion, we summarize the synthesis, structure, and corresponding BTE applications of Bio‐RGMs, as shown in **Table** **4**.

**Table 4:** Summary of the synthesis, structure, and applications of Bio‐RGMs in BTE.

Bio‐RGMs	Synthesis method	Structure	Application	References
SerMA/GO	Photo‐crosslinking	3D scaffold	Bone regeneration	[124]
CS/RGO	3D printing	3D scaffold	Bone tissue regeneration	[125]
PDA/GO/COL	Self‐assembly	2D membrane	BTE	[126]
PLGA/β‐TCP/GO	3D printing	3D scaffold	Bone repair	[127]
Fibrin hydrogel/GO	Templated synthesis	3D scaffold	Bone repair	[128]
GO/alginate/gelatin	3D bioprinting	3D scaffold	Bone tissue models	[129]
GO/SF/nHA	Cross‐linking and freeze‐drying	3D scaffold	Bone regeneration	[130]
GO/peptide	Self‐assembly	nanohybrid	Osteogenesis of hAMSCs	[131]
GP‐peptide‐HA	Noncovalent linking	2D paper	Osteogenesis of BMSCs	[30]
GO/elastin	Covalent linking	2D membrane	Bone regeneration	[120]
PAN/PEG‐MgO/g‐C3N4	Electrospinning	Membrane	Bone regeneration	[132]
Peptide/*m*‐GO	Self‐assembly	2D film	Osteogenic differentiation	[133]
GO‐GelMA‐Ap	Cross‐linking	Microgels	Bone repair	[134]
GO/alginate	3D printing	3D scaffold	Bone regeneration	[137]
PLA/PDA/RGO	3D printing	3D scaffold	Bone grafting	[138]
PC/GO	Electrospinning	3D scaffold	Bone grafting	[140]
PCL/CS/COL/GO	Electrospinning	3D scaffold	Bone grafting	[141]
COL‐RGO	Chemical cross‐linking and freeze‐drying	3D scaffold	Bone regeneration	[142]
GO/COL	Chemical cross‐linking and freeze‐drying	Aerogels	Bone regeneration	[143]
GO/COL	3D printing	3D scaffold	BTE	[144]
CS/GO	Chemical cross‐linking and freeze‐drying	3D porous scaffold	Drug delivery and bone regeneration	[145]
BMP2/GO/SF	Electrospinning	Scaffold	Drug nanocarriers	[146]
BMP2/GO/PLGA	Electrospinning	3D scaffold	Calvarial defect repair	[147]
GO/BFP/PCL	Electrospinning	3D scaffold	Cell delivery	[148]
VauHp@RGO	Chemical linking	composites	Bone regeneration	[149]
IGF@C3N4/NGF@SF	Electrospinning	fibers	BTE	[150]
GO/ALG/GEL	3D printing	Scaffold	Loading bone cells	[129]
RGO/GelMA	3D printing	Hydrogels	Loading cells	[72]
GEL/ALG/GO	Chemical cross‐linking and freeze‐drying	Scaffold	Bone regeneration	[151]
G/GO/PCL	3D printing	3D scaffold	Cancer therapy and bone regeneration	[152]
GG/GO/Cur	3D printing	3D scaffold	Tumor treatment and bone reconstruction	[153]
MgNPs/GNSs	Adsorption and self‐assembly	Nanoscrolls	Inflammatory response	[154]

**Figure 12:**
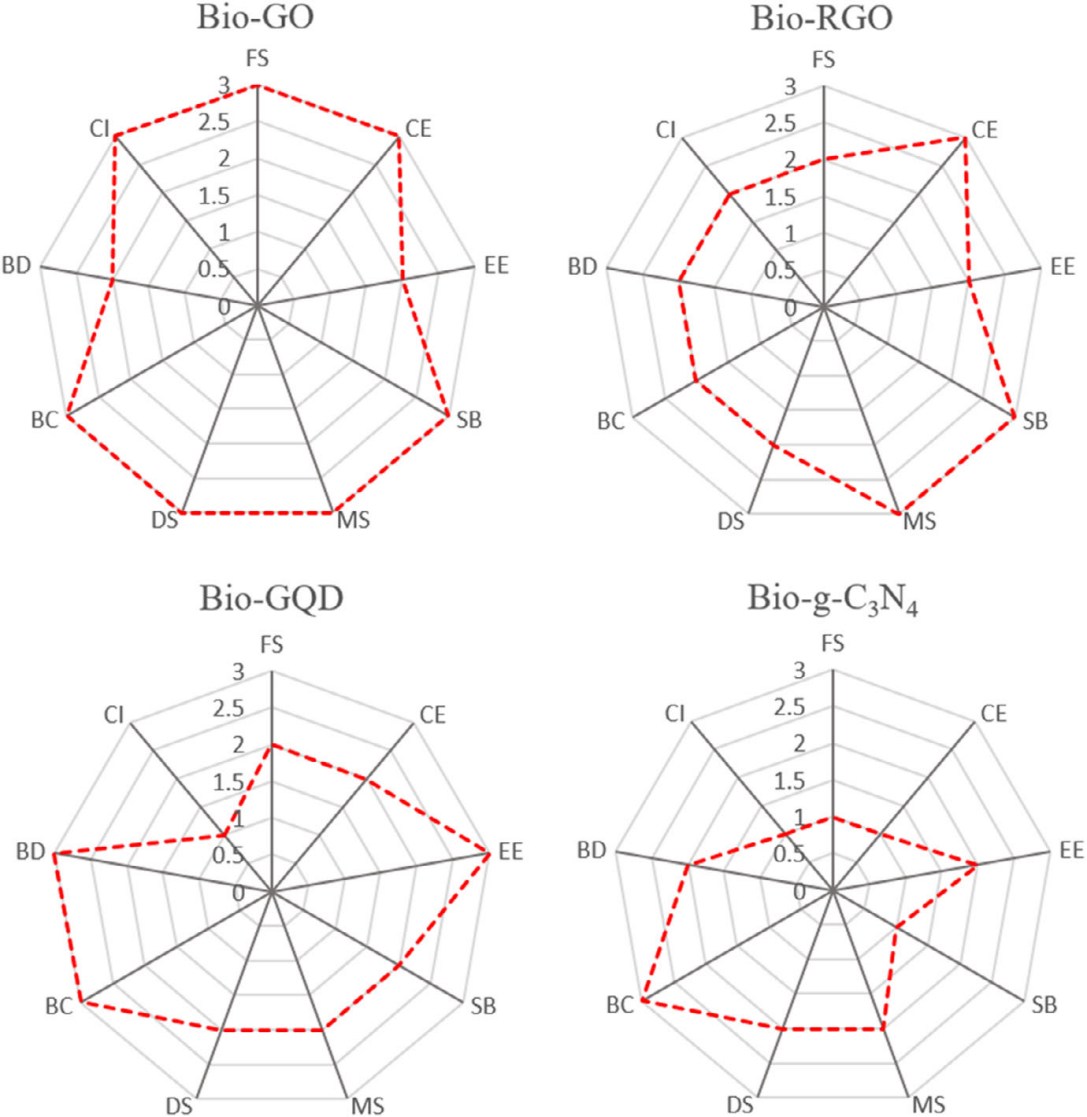
Factor analysis of several Bio‐RGMs for BTE applications.

To apply designed Bio‐RGMs for practical BTE applications, some factors of the Bio‐RGMs should be considered, including at least the (FS) feasibility of synthesis, (CE) cost‐effectivity, (EE) environmental effect, (SB) stability, (MS) mechanical strength, (DB) degree of biomodification, (BC) biocompatibility, (BD) biodegradability, and (CI) cellular induction. By considering these factors, we further analyze the potential of 4 main types of Bio‐RGMs (Bio‐GO, Bio‐RGO, Bio‐GQD, and Bio‐g‐C_3_N_4_) toward BTE applications. To simplify the analysis, we compare only these 4 Bio‐RGMs and give a value (high = 3, medium = 2, and low = 1) for all these factors. For instance, for the factor of FS, the values of 3, 2, 2, and 1 are used for Bio‐GO, Bio‐RGO, Bio‐GQD, and Bio‐g‐C_3_N_4_, respectively; for the factor of CE, 3, 3, 2, and 1 are given. **Figure** **12** presents the factor analysis of Bio‐GO, Bio‐RGO, Bio‐GQD, and Bio‐g‐C_3_N_4_ that are related to BTE applications. It can be found that GO‐based Bio‐RGMs exhibited the advantages for using in BTE applications than RGO, GQDs, and g‐C_3_N_4_.

Although various GMs and Bio‐RGMs have been widely studied and applied in fields such as biosensing, drug delivery, BTE, regenerative medicine, cancer therapy, and bioimaging, their actual clinical use remains limited. Two main challenges persist: ensuring the long‐term biological safety of Bio‐RGMs for the human body, and achieving low‐cost, large‐scale, high‐quality production of graphene materials to meet the demands of future clinical studies.

## 6 Conclusion and Perspectives

In this review, we have highlighted recent advancements in the design and synthesis of Bio‐RGMs for BTE applications. We emphasized the critical role of interactions between GMs and biomolecules in facilitating both covalent and noncovalent bioconjugation. Various techniques, including electrospinning, 3D printing, self‐assembly, templated synthesis, freeze‐drying, and others, enabled the tailoring of Bio‐RGM structures, ranging from nanohybrids to 1D nanofibers, 2D membranes, and 3D scaffolds/hydrogels/aerogels. Bio‐RGMs, particularly 2D membranes and 3D structures, serve as excellent matrices for delivering drugs, growth factors, and stem cells into bone defects, thereby promoting bone growth, repair, and regeneration. Through molecular design and biomimetic properties, Bio‐RGMs exhibited enhanced biocompatibility, bioactivity, biotargeting, and antibacterial activity, expanding their applications in bone repair/regeneration, bone grafting, drug‐assisted bone growth, and bone tumor inhibition. This review provides valuable insights and key considerations in the field of Bio‐RGM research, aiming to inspire the development of novel Bio‐RGMs for tissue engineering and biomedical applications.

In this promising research field, there are several challenges that should be addressed. First, although the bioengineering of GMs with biomolecules has reduced the toxicity of materials to cells and tissues, the long‐term biocompatibility and cytotoxicity of Bio‐RGMs should be considered after the degradation or release of biomolecules from the materials. Therefore, further study is needed for developing novel functionalization methods and techniques to uncover the molecule‐material interactions. Second, while previous studies have focused on the functional aspects of materials and their effects on bone cells, there is relatively limited research on the action mechanisms of Bio‐RGMs and their biological effects. It is suggested that an in‐depth investigation into the biological interaction mechanism and the structure‐efficiency relationship of Bio‐RGMs could be conducted. Third, besides GMs, other 2D materials such as MXene and black phosphorus can be combined with functional biomolecules to synthesize photo‐sensitive hybrid materials, which could exhibit photothermal, photodynamic, and antibacterial effects on cells and tissues, which are also interesting for BTE applications. Finally, while much attention is currently directed toward basic research on materials synthesis and preliminary applications, there is a pressing need for practical clinical utilization of Bio‐RGMs in BTE. Highly precise and effective fabrication techniques are necessary to prepare implant products that meet the requirements of different cases.

## Conflict of Interest

The authors declare no conflict of interest.

## Author Contributions

**Dagang Li**: Data curation (equal); Formal analysis (equal); Investigation (lead); Methodology (lead); Software (equal); Writing—original draft (lead). **Jinze Zhao**: Data curation (supporting); Formal analysis (supporting); Investigation (supporting); Methodology (supporting); Software (supporting); Writing—original draft (supporting). **Yuan Wang**: Data curation (supporting); Formal analysis (supporting); Resources (supporting); Software (supporting); Writing—original draft (supporting). **Jialu Wang**: Data curation (supporting); Formal analysis (supporting); Resources (supporting); Software (supporting). **Zhenjuan Sun**: Data curation (supporting); Investigation (supporting); Methodology (supporting); Software (supporting). **Fuxin Wei**: Conceptualization (equal); Funding acquisition (equal); Project administration (equal); Supervision (supporting); Writing—review & editing (supporting). **Gang Wei**: Conceptualization (equal); Funding acquisition (equal); Project administration (equal); Supervision (equal); Writing—review & editing (equal). **Zhengang Sun**: Conceptualization (supporting); Funding acquisition (equal); Project administration (equal); Supervision (equal); Writing—review & editing (equal).

## References

[1] H. B. Huang , H. D. Shi , P. Das , J. Q. Qin , Y. G. Li , X. Wang , F. Su , P. C. Wen , S. Y. Li , P. F. Lu , F. Y. Liu , Y. J. Li , Y. Zhang , Y. Wang , Z. S. Wu , H. M. Cheng , Adv. Funct. Mater. 2020, 30, 1909035.

[2] F. R. Fan , R. X. Wang , H. Zhang , W. Z. Wu , Chem. Soc. Rev. 2021, 50, 10983. 34617521 10.1039/c9cs00821g

[3] A. Stergiou , R. Cantón‐Vitoria , M. N. Psarrou , S. P. Economopoulos , N. Tagmatarchis , Prog. Mater. Sci. 2020, 114, 100683.

[4] S. G. Wang , X. Q. Yang , L. L. Zhou , J. F. Li , H. R. Chen , J. Mater. Chem. B 2020, 8, 2974. 32207478 10.1039/c9tb02845e

[5] C. Martín , K. Kostarelos , M. Prato , A. Bianco , Chem. Commun. 2019, 55, 5540. 10.1039/c9cc01205b 31033990

[6] C. J. Bullock , C. Bussy , Adv. Mater. Interfaces 2019, 6, 1900229.

[7] M. Tahriri , M. Del Monico , A. Moghanian , M. T. Yaraki , R. Torres , A. Yadegari , L. Tayebi , Mater. Sci. Eng., C 2019, 102, 171. 10.1016/j.msec.2019.04.051 31146988

[8] M. Adeel , M. Bilal , T. Rasheed , A. Sharma , H. M. N. Iqbal , Int. J. Biol. Macromol. 2018, 120, 1430. 30261251 10.1016/j.ijbiomac.2018.09.144

[9] K. R. Nandanapalli , D. Mudusu , S. Lee , Carbon 2019, 152, 954.

[10] F. C. Wang , K. D. Wang , B. X. Zheng , X. Dong , X. S. Mei , J. Lv , W. Q. Duan , W. J. Wang , Mater. Technol. 2018, 33, 340.

[11] Y. T. Wang , S. H. Di , J. H. Yu , L. Wang , Z. Li , J. Mater. Chem. B 2023, 11, 500. 36541392 10.1039/d2tb01962k

[12] D. P. Li , W. S. Zhang , X. Q. Yu , Z. P. Wang , Z. Q. Su , G. Wei , Nanoscale 2016, 8, 19491. 27878179 10.1039/c6nr07249f

[13] X. Q. Zou , S. Wei , J. Jasensky , M. Y. Xiao , Q. M. Wang , C. L. Brooks , Z. Chen , J. Am. Chem. Soc. 2017, 139, 1928. 28092440 10.1021/jacs.6b11226

[14] P. Passaretti , Front. Mol. Biosci. 2022, 9, 774097. 35372519 10.3389/fmolb.2022.774097 PMC8965154

[15] L. Wang , Y. J. Zhang , A. G. Wu , G. Wei , Anal. Chim. Acta 2017, 985, 24. 28864192 10.1016/j.aca.2017.06.054

[16] S. S. Sekhon , P. Kaur , Y. H. Kim , S. S. Sekhon , NPJ 2D Mater. Appl. 2021, 5, 21.

[17] Z. B. Qu , X. U. Zhou , M. Zhang , J. L. Shen , Q. Li , F. Xu , N. Kotov , C. H. Fan , Adv. Mater. 2021, 33, 2007900. 10.1002/adma.202007900 33960020

[18] S. Kaushal , A. Kumar , S. Soni , N. K. Singhal , Sens. Actuators, B 2021, 329, 129141.

[19] S. Joshi , R. Siddiqui , P. Sharma , R. Kumar , G. Verma , A. Saini , Sci. Rep. 2020, 10, 9441. 32523022 10.1038/s41598-020-66230-3 PMC7287048

[20] B. Liu , P. He , H. Kong , D. Z. Zhu , G. Wei , Macromol. Mater. Eng. 2022, 307, 2100886.

[21] H. Q. Zhu , B. Zhang , N. L. Zhu , M. C. Li , Q. L. Yu , Chin. Chem. Lett. 2021, 32, 1220.

[22] C. Ligorio , M. O’Brien , N. W. Hodson , A. Mironov , M. Iliut , A. F. Miller , A. Vijayaraghavan , J. A. Hoyland , A. Saiani , Acta Biomater. 2021, 127, 116. 33831573 10.1016/j.actbio.2021.03.077

[23] X. T. Wang , W. M. Guo , L. Y. Li , F. Yu , J. Y. Li , L. Liu , B. Fang , L. G. Xia , Chem. Eng. J. 2021, 413, 127413.

[24] S. D. Newby , T. Masi , C. D. Griffin , W. J. King , A. Chipman , S. Stephenson , D. E. Anderson , A. S. Biris , S. E. Bourdo , M. Dhar , Int. J. Nanomed. 2020, 15, 2501. 10.2147/IJN.S245801 PMC7171876 32368037

[25] B. Guo , X. D. Feng , Y. Wang , X. S. Wang , Y. He , J. Mater. Chem. B 2021, 9, 9720. 34787627 10.1039/d1tb00618e

[26] H. Shen , H. Lin , A. X. Sun , S. J. Song , B. Wang , Y. H. Yang , J. W. Dai , R. S. Tuan , Acta Biomater. 2020, 105, 44. 32035282 10.1016/j.actbio.2020.01.048

[27] Y. C. Gao , K. Kang , B. Luo , X. Q. Sun , F. Lan , J. He , Y. Wu , Regener. Biomater. 2022, 9, rbac024. 10.1093/rb/rbac024 PMC9071057 35529047

[28] N. Amiryaghoubi , N. N. Pesyan , M. Fathi , Y. Omidi , Int. J. Biol. Macromol. 2020, 162, 1338. 32561280 10.1016/j.ijbiomac.2020.06.138

[29] Y. J. Shuai , C. B. Mao , M. Y. Yang , ACS Appl. Mater. Interfaces 2018, 10, 31988. 30204402 10.1021/acsami.8b11811 PMC6310480

[30] M. J. Wang , T. Yang , Q. Bao , M. Y. Yang , C. B. Mao , ACS Appl. Mater. Interfaces 2022, 14, 350. 34962367 10.1021/acsami.1c20740

[31] R. G. Bai , N. Ninan , K. Muthoosamy , S. Manickam , Prog. Mater. Sci. 2018, 91, 24.

[32] L. Daneshmandi , M. Barajaa , A. T. Rad , S. A. Sydlik , C. T. Laurencin , Adv. Healthcare Mater. 2021, 10, 2001414. 10.1002/adhm.202001414 PMC8218309 33103370

[33] F. Edrisi , N. Baheiraei , M. Razavi , K. Roshanbinfar , R. Imani , N. Jalilinejad , J. Mater. Chem. B 2023, 11, 7280. 37427687 10.1039/d3tb00654a

[34] R. G. Bai , K. Muthoosamy , S. Manickam , A. Hilal‐Alnaqbi , Int. J. Nanomed. 2019, 14, 5753. 10.2147/IJN.S192779 PMC6662516 31413573

[35] R. Guazzo , C. Gardin , G. Bellin , L. Sbricoli , L. Ferroni , F. S. Ludovichetti , A. Piattelli , I. Antoniac , E. Bressan , B. Zavan , Nanomaterials 2018, 8, 349. 29783786 10.3390/nano8050349 PMC5977363

[36] M. de Sousa , C. H. Z. Martins , L. S. Franqui , L. C. Fonseca , F. S. Delite , E. M. Lanzoni , D. S. T. Martinez , O. L. Alves , J. Mater. Chem. B 2018, 6, 2803. 32254233 10.1039/c7tb02997g

[37] H. Seelajaroen , A. Bakandritsos , M. Otyepka , R. Zboril , N. S. Sariciftci , ACS Appl. Mater. Interfaces 2020, 12, 250. 31816230 10.1021/acsami.9b17777 PMC6953471

[38] J. Jagiello , M. Kusmierz , E. Kijenska‐Gawronska , M. Winkowska‐Struzik , W. Swieszkowski , L. Lipinska , Mater. Today Commun. 2021, 26, 102056.

[39] H. N. Farrag , A. L. T. Zheng , S. Sabidi , Y. Wu , S. Ikeno , T. Maeda , Y. Andou , T. Kato , Int. J. Environ. Sci. Technol. 2024, 21, 1481.

[40] L. Zhou , K. Wang , H. Sun , S. M. Zhao , X. F. Chen , D. H. Qian , H. J. Mao , J. L. Zhao , Nano‐Micro Lett. 2019, 11, 20. 10.1007/s40820-019-0250-8 PMC7770693 34137997

[41] G. Wei , Y. Zhang , S. Steckbeck , Z. Q. Su , Z. Li , J. Mater. Chem. 2012, 22, 17190.

[42] M. Tian , M. Qiao , C. C. Shen , F. L. Meng , L. A. Frank , V. V. Krasitskaya , T. J. Wang , X. M. Zhang , R. H. Song , Y. X. Li , J. J. Liu , S. C. Xu , J. H. Wang , Appl. Surf. Sci. 2020, 527, 146839.

[43] K. E. Eckhart , B. D. Holt , M. G. Laurencin , S. A. Sydlik , Biomater. Sci. 2019, 7, 3876. 31309944 10.1039/c9bm00867e

[44] A. Serrano‐Aroca , L. Iskandar , S. Deb , Eur. Polym. J. 2018, 103, 198.

[45] J. H. Wang , X. J. Zhao , J. F. Li , X. Kuang , Y. Q. Fan , G. Wei , Z. Q. Su , ACS Macro Lett. 2014, 3, 529. 35590718 10.1021/mz500213w

[46] Y. B. Xiong , Z. H. Lu , D. D. Wang , M. N. O. Yang , H. M. Guo , Z. H. Yang , J. Chromatogr. A 2020, 1614, 460725. 31767260 10.1016/j.chroma.2019.460725

[47] U. Farooq , J. G. Zhuang , X. H. Wang , S. G. Lyu , Chem. Eng. J. 2021, 403, 126405.

[48] H. J. Zhang , A. R. Ding , B. T. Ye , Z. Q. Wang , J. W. Zhang , L. P. Qiu , J. H. Chen , ACS Appl. Nano Mater. 2021, 4, 8546.

[49] Y. J. Wu , T. V. Tam , P. H. Rao , W. M. Choi , I. K. Yoo , Appl. Surf. Sci. 2024, 654, 159490.

[50] L. Zhang , Y. B. Sheng , A. Z. Yazdi , K. Sarikhani , F. Wang , Y. S. Jiang , J. W. Liu , T. Zheng , W. Wang , P. K. Ouyang , P. Chen , Nanoscale 2019, 11, 2999. 30698183 10.1039/c8nr08397e

[51] D. Z. Zhu , P. He , H. Kong , G. Z. Yang , X. Luan , G. Wei , J. Mater. Chem. B 2022, 10, 9216. 36314985 10.1039/d2tb02132c

[52] F. Li , W. J. Ma , J. C. Liu , X. Wu , Y. Wang , J. B. He , Anal. Bioanal. Chem. 2018, 410, 543. 29167935 10.1007/s00216-017-0752-5

[53] X. Li , L. Q. Yang , Y. F. Wang , Z. Y. Du , X. Y. Mao , D. Z. Sun , J. Liu , Y. Zhou , X. Y. Xu , IET Nanobiotechnol. 2020, 14, 308. 32463021 10.1049/iet-nbt.2019.0377 PMC8676041

[54] S. Husale , S. Sahoo , A. Radenovic , F. Traversi , P. Annibale , A. Kis , Langmuir 2010, 26, 18078. 20977263 10.1021/la102518t

[55] A. Baek , Y. M. Baek , H. M. Kim , B. H. Jun , D. E. Kim , Bioconjugate Chem. 2018, 29, 528. 10.1021/acs.bioconjchem.8b00025 29376329

[56] Y. Li , P. P. Zhang , Z. F. Ouyang , M. F. Zhang , Z. J. Lin , J. F. Li , Z. Q. Su , G. Wei , Adv. Funct. Mater. 2016, 26, 2122.

[57] G. P. Awasthi , V. K. Kaliannagounder , J. Park , B. Maharjan , M. Shin , C. Yu , C. H. Park , C. S. Kim , Colloids Surf., A 2021, 622, 126584.

[58] H. S. Budi , M. J. Ansari , S. A. Jasim , W. K. Abdelbasset , D. Bokov , Y. F. Mustafa , M. A. A. Najm , M. Kazemnejadi , Inorg. Chem. Commun. 2022, 139, 109336.

[59] W. Su , Z. Y. Wang , J. Jiang , X. Y. Liu , J. Z. Zhao , Z. J. Zhang , Int. J. Nanomed. 2019, 14, 1835. 10.2147/IJN.S183842 PMC6417852 30880983

[60] S. D. Liu , Z. R. Li , Q. X. Wang , J. Han , W. Y. Wang , S. H. Li , H. F. Liu , S. T. Guo , J. C. Zhang , K. Ge , G. Q. Zhou , ACS Appl. Bio Mater. 2021, 4, 8049. 10.1021/acsabm.1c00967 35006786

[61] F. Ghorbani , A. Zamanian , A. Aidun , J. Appl. Polym. Sci. 2019, 136, 47656.

[62] Y. Chen , G. Z. Yang , B. Liu , H. Kong , Z. Xiong , L. Guo , G. Wei , Chem. Eng. J. 2022, 430, 132721.

[63] P. He , M. H. Yang , Y. Lei , L. Guo , Y. Wang , G. Wei , Polymers 2023, 15, 1321. 36904561 10.3390/polym15051321 PMC10006990

[64] K. H. Li , Z. F. Zhang , D. P. Li , W. S. Zhang , X. Q. Yu , W. Liu , C. C. Gong , G. Wei , Z. Q. Su , Adv. Funct. Mater. 2018, 28, 1801056.

[65] I. H. Ali , A. Ouf , F. Elshishiny , M. B. Taskin , J. Song , M. D. Dong , M. L. Chen , R. Siam , W. Mamdouh , ACS Omega 2022, 7, 1838. 35071876 10.1021/acsomega.1c05095 PMC8771952

[66] W. Wang , J. R. Passarini , P. R. L. Nalesso , D. Musson , J. Cornish , F. Mendonça , G. F. Caetano , P. Bártolo , Mater. Sci. Eng., C 2019, 100, 759. 10.1016/j.msec.2019.03.047 30948113

[67] G. Z. Yang , P. He , D. Z. Zhu , K. M. Wan , H. Kong , X. Luan , L. Fang , Y. Wang , G. Wei , Environ. Sci. Nano 2022, 9, 4497.

[68] S. Asha , A. N. Ananth , S. P. Jose , M. A. J. Rajan , Appl. Nanosci. 2018, 8, 395.

[69] S. Asha , G. V. Kumar , A. N. Ananth , S. P. Jose , M. A. J. Rajan , Mater. Today Proc. 2019, 9, 389.

[70] C. Ligorio , M. Zhou , J. K. Wychowaniec , X. Y. Zhu , C. Bartlam , A. F. Miller , A. Vijayaraghavan , J. A. Hoyland , A. Saiani , Acta Biomater. 2019, 92, 92. 31091473 10.1016/j.actbio.2019.05.004 PMC6582688

[71] X. Y. Ding , Y. R. Yu , L. R. Shang , Y. J. Zhao , ACS Nano 2022, 16, 19533. 36269119 10.1021/acsnano.2c09850

[72] X. W. Zhang , H. Zhang , Y. Zhang , H. M. Huangfu , Y. X. Yang , Q. Y. Qin , Y. D. Zhang , Y. M. Zhou , J. Mater. Chem. B 2023, 11, 1288. 36651822 10.1039/d2tb01979e

[73] F. Tian , J. Lyu , J. Y. Shi , M. Yang , Biosens. Bioelectron. 2017, 89, 123. 27342369 10.1016/j.bios.2016.06.046

[74] S. Rahimi , Y. Y. Chen , M. Zareian , S. Pandit , I. Mijakovic , Adv. Drug Delivery Rev. 2022, 189, 114467. 10.1016/j.addr.2022.114467 35914588

[75] C. Park , S. Park , D. Lee , K. S. Choi , H. P. Lim , J. Kim , Tissue Eng. Regener. Med. 2017, 14, 481. 10.1007/s13770-017-0052-3 PMC6171627 30603503

[76] S. Sattari , M. Adeli , S. Beyranvand , M. Nemati , Int. J. Nanomed. 2021, 16, 5955. 10.2147/IJN.S249712 PMC8416335 34511900

[77] L. F. Madeo , P. Sarogni , G. Cirillo , O. Vittorio , V. Voliani , M. Curcio , T. Shai‐Hee , B. Büchner , M. Mertig , S. Hampel , Materials 2022, 15, 1648. 35268879 10.3390/ma15051648 PMC8911244

[78] X. L. Ding , H. F. Liu , Y. B. Fan , Adv. Healthcare Mater. 2015, 4, 1451.

[79] V. Srimaneepong , H. E. Skallevold , Z. Khurshid , M. S. Zafar , D. Rokaya , J. Sapkota , Int. J. Mol. Sci. 2022, 23, 499. 35008923 10.3390/ijms23010499 PMC8745297

[80] H. Xie , T. Cao , F. J. Rodríguez‐Lozano , E. K. Luong‐Van , V. Rosa , Dent. Mater. 2017, 33, 765. 28495017 10.1016/j.dental.2017.04.008

[81] S. K. Krishnan , E. Singh , P. Singh , M. Meyyappan , H. S. Nalwa , RSC Adv. 2019, 9, 8778. 35517682 10.1039/c8ra09577a PMC9062009

[82] M. E. V. Zapata , C. D. G. Tovar , J. H. M. Hernandez , Biomolecules 2020, 10, 1616. 33265973 10.3390/biom10121616 PMC7760599

[83] J. Kang , V. T. Nguyen , M. S. Kim , Anal. Chem. 2023, 95, 9505. 37310094 10.1021/acs.analchem.3c00647 PMC10308326

[84] K. Chauhan , J. Woo , W. Jung , D. E. Kim , Materials 2023, 16, 7434. 38068178 10.3390/ma16237434 PMC10707405

[85] J. L. Aparicio‐Collado , N. García‐San‐Martín , J. Molina‐Mateo , C. T. Cabanilles , V. D. Quiles , A. Serrano‐Aroca , R. S. I. Serra , Colloids Surf., B 2022, 214, 112455. 10.1016/j.colsurfb.2022.112455 35305322

[86] Q. Yu , C. T. Shen , X. S. Wang , Z. X. Wang , L. Liu , J. F. Zhang , Int. J. Nanomed. 2023, 18, 563. 10.2147/IJN.S392782 PMC9900644 36756050

[87] Z. M. Markovic , S. P. Jovanovic , P. Z. Maskovic , M. Danko , M. Micusík , V. B. Pavlovic , D. D. Milivojevic , A. Kleinová , Z. Spitalsky , B. M. T. Markovic , RSC Adv. 2018, 8, 31337. 35548242 10.1039/c8ra04664f PMC9085601

[88] S. Pandit , K. Gaska , R. Kádár , I. Mijakovic , ChemPhysChem 2021, 22, 250. 33244859 10.1002/cphc.202000769 PMC7898826

[89] A. G. Williams , E. Moore , A. Thomas , J. A. Johnson , Int. J. Biomater. 2023, 2023, 8803283. 36819211 10.1155/2023/8803283 PMC9929215

[90] A. Shariati , S. M. Hosseini , Z. Chegini , A. Seifalian , M. R. Arabestani , Biomed. Pharmacother. 2023, 158, 114184. 36587554 10.1016/j.biopha.2022.114184

[91] S. Kulshrestha , S. Khan , R. Meena , B. R. Singh , A. U. Khan , Biofouling 2014, 30, 1281. 25431994 10.1080/08927014.2014.983093

[92] M. Y. Mao , W. J. Zhang , Z. W. Huang , J. Huang , J. Wang , W. P. Li , S. S. Gu , Int. J. Nanomed. 2021, 16, 7727. 10.2147/IJN.S303521 PMC8610231 34824531

[93] N. Baheiraei , M. Razavi , R. Ghahremanzadeh , Biomater. Res. 2023, 27, 109. 37924106 10.1186/s40824-023-00449-9 PMC10625265

[94] A. Moeinzadeh , B. Ashtari , H. Garcia , M. Koruji , C. A. Velazquez , Z. Bagher , M. Barati , R. Shabani , S. M. Davachi , J. Funct. Biomater. 2023, 14, 556. 38132810 10.3390/jfb14120556 PMC10744091

[95] F. Xie , L. Y. Zou , Z. K. Xu , X. L. Ou , W. L. Guo , Y. Gao , G. H. Gao , Int. J. Biol. Macromol. 2022, 223, 391. 36356865 10.1016/j.ijbiomac.2022.11.013

[96] Y. H. Bao , H. H. Li , J. He , K. Song , H. Z. Yu , C. L. Tian , J. Guo , X. W. Zhou , S. M. Liu , Colloids Surf., B 2023, 229, 113435. 10.1016/j.colsurfb.2023.113435 37437413

[97] W. A. Khalil , H. H. A. Sherif , B. A. Hemdan , S. K. H. Khalil , W. E. I. Hotaby , IET Nanobiotechnol. 2019, 13, 816. 31625521 10.1049/iet-nbt.2018.5321 PMC8676512

[98] S. Mohammadi , A. Babaei , Int. J. Biol. Macromol. 2022, 201, 528. 35051501 10.1016/j.ijbiomac.2022.01.086

[99] M. Kern , S. Skulj , M. Rozman , Chemosphere 2022, 296, 134010. 35181425 10.1016/j.chemosphere.2022.134010

[100] S. R. Tan , X. Wu , Y. Q. Xing , S. Lilak , M. Wu , J. X. Zhao , Colloids Surf., B 2020, 185, 110616. 10.1016/j.colsurfb.2019.110616 31740323

[101] J. Wu , C. M. Wang , S. S. Zhang , L. Zhang , J. S. Hao , Z. J. Jia , X. M. Zheng , Y. G. Lv , S. Fu , G. L. Zhang , Micromachines 2024, 15, 122.38258241

[102] W. J. Cao , L. He , W. D. Cao , X. B. Huang , K. Jia , J. Y. Dai , Acta Biomater. 2020, 112, 14. 32531395 10.1016/j.actbio.2020.06.009

[103] N. Mauro , C. Scialabba , S. Agnello , G. Cavallaro , G. Giammona , Mater. Sci. Eng., C 2020, 107, 110201. 10.1016/j.msec.2019.110201 31761243

[104] C. Xu , H. Hong , Y. Lee , K. S. Park , M. J. Sun , T. R. Wang , M. E. Aikins , Y. Xu , J. J. Moon , ACS Nano 2020, 14, 13268. 32902245 10.1021/acsnano.0c05062 PMC7606610

[105] Y. Yin , X. Y. Li , H. X. Ma , J. Zhang , D. Yu , R. F. Zhao , S. J. Yu , G. J. Nie , H. Wang , Nano Lett. 2021, 21, 2224. 33594887 10.1021/acs.nanolett.0c05039

[106] Z. Abdollahi , A. Taheri‐Kafrani , S. A. Bahrani , A. A. Kajani , J. Biotechnol. 2019, 298, 88. 30986517 10.1016/j.jbiotec.2019.04.006

[107] J. Charmi , H. Nosrati , J. M. Amjad , R. Mohammadkhani , H. Danafar , Heliyon 2019, 5, e01466. 31011643 10.1016/j.heliyon.2019.e01466 PMC6460424

[108] P. Maleki , A. Dinari , B. Jahangiri , J. Raheb , PLoS One 2023, 18, e0295822. 38096179 10.1371/journal.pone.0295822 PMC10720998

[109] L. L. Ou , T. Sun , M. Y. Liu , Y. Zhang , Z. Y. Zhou , X. Z. Zhan , L. H. Lu , Q. T. Zhao , R. F. Lai , L. Q. Shao , Int. J. Nanomed. 2020, 15, 1569. 10.2147/IJN.S220057 PMC7069571 32210552

[110] J. Liu , C. N. Li , T. Brans , A. Harizaj , S. Van de Steene , T. De Beer , S. De Smedt , S. Szunerits , R. Boukherroub , R. H. Xiong , K. Braeckmans , Int. J. Mol. Sci. 2020, 21, 1540. 32102402 10.3390/ijms21041540 PMC7073198

[111] B. Vakili , M. Karami‐Darehnaranji , E. Mirzaei , F. Hosseini , N. Nezafat , Int. Immunopharmacol. 2023, 125, 111062. 37866317 10.1016/j.intimp.2023.111062

[112] S. Y. Huang , Y. Y. Li , S. Zhang , Y. M. Chen , W. Q. Su , D. J. Sanchez , J. D. H. Mai , X. Zhi , H. J. Chen , X. T. Ding , J. Controlled Release 2024, 365, 716. 10.1016/j.jconrel.2023.11.047 38036004

[113] Q. Y. Bai , Z. W. Wang , A. N. Yina , J. J. Tian , Z. L. Li , Y. F. Yang , Y. J. Dong , M. Y. Chen , T. L. Liu , Vaccine 2022, 40, 7613. 36371365 10.1016/j.vaccine.2022.11.005

[114] Q. Xiang , J. Y. Huang , H. Y. Huang , W. W. Mao , Z. Z. Ye , RSC Adv. 2018, 8, 1820. 35542626 10.1039/c7ra11945c PMC9077103

[115] M. Chaturvedi , M. Patel , N. Bisht , M. D. Mukherjee , A. Tiwari , D. P. Mondal , A. K. Srivastava , N. Dwivedi , C. Dhand , Biosensors 2023, 13, 342. 36979554 10.3390/bios13030342 PMC10046000

[116] G. M. Ji , J. K. Tian , F. Xing , Y. Feng , Int. J. Mol. Sci. 2022, 23, 10838.36142748

[117] K. W. Chen , L. B. Chen , Y. Q. Chen , H. Bai , L. Li , J. Mater. Chem. 2012, 22, 20968.

[118] X. Huang , X. Y. Qi , F. Boey , H. Zhang , Chem. Soc. Rev. 2012, 41, 666. 21796314 10.1039/c1cs15078b

[119] A. Balaji , S. L. Yang , J. Wang , J. Zhang , Biosensors 2019, 9, 74.31151203

[120] H. Li , E. Buck , O. Elkashty , S. D. Tran , T. Szkopek , M. Cerruti , ACS Appl. Nano Mater. 2022, 5, 6890.

[121] S. Park , Y. K. Kim , S. Kim , B. Son , J. Jang , T. H. Park , Biomater. Adv. 2023, 144, 213221. 36459949 10.1016/j.bioadv.2022.213221

[122] E. S. Kang , D. S. Kim , I. R. Suhito , S. S. Choo , S. J. Kim , I. Song , T. H. Kim , Nano Convergence 2017, 4, 2. 28191446 10.1186/s40580-017-0096-z PMC5271168

[123] D. L. Jiao , A. Zheng , Y. Liu , X. K. Zhang , X. Wang , J. N. Wu , W. J. She , K. G. Lv , L. Y. Cao , X. Q. Jiang , Bioact. Mater. 2021, 6, 2011. 33426373 10.1016/j.bioactmat.2020.12.003 PMC7782557

[124] C. Qi , Y. Deng , L. M. Xu , C. Yang , Y. Y. Zhu , G. B. Wang , Z. Wang , L. Wang , Theranostics 2020, 10, 741. 31903148 10.7150/thno.39502 PMC6929981

[125] C. S. D. Cabral , S. P. Miguel , D. de Melo‐Diogo , R. O. Louro , I. J. Correia , Carbon 2019, 146, 513.

[126] S. Davaie , T. Hooshmand , F. Najafi , M. H. Nazarpak , M. Pirmoradian , ACS Appl. Bio Mater. 2023, 6, 4629. 10.1021/acsabm.3c00400 37930634

[127] Y. D. Zhang , C. Wang , L. Fu , S. Ye , M. Wang , Y. M. Zhou , Molecules 2019, 24, 1669. 31035401 10.3390/molecules24091669 PMC6539066

[128] S. Pathmanapan , P. Periyathambi , S. K. Anandasadagopan , Nanomed. Nanotechnol. Bio. Med. 2020, 29, 102251. 10.1016/j.nano.2020.102251 32592758

[129] J. H. Zhang , H. Eyisoylu , X. H. Qin , M. Rubert , R. Müller , Acta Biomater. 2021, 121, 637. 33326888 10.1016/j.actbio.2020.12.026

[130] J. C. Sun , L. Li , F. Xing , Y. Yang , M. Gong , G. M. Liu , S. Wu , R. Luo , X. Duan , M. Liu , M. Zou , Z. Xiang , Stem Cell Res. Ther. 2021, 12, 591. 34863288 10.1186/s13287-021-02634-w PMC8642892

[131] E. S. Kang , H. Kim , Y. Han , Y. W. Cho , H. Son , Z. T. Luo , T. H. Kim , Colloids Surf., B 2021, 204, 111807. 10.1016/j.colsurfb.2021.111807 33964530

[132] B. Danagody , N. Bose , K. Rajappan , A. Iqbal , G. M. Ramanujam , A. K. Anilkumar , ACS Biomater. Sci. Eng. 2023, 10, 468. 38078836 10.1021/acsbiomaterials.3c00892

[133] P. Y. Puah , P. Y. Moh , C. S. Sipaut , P. C. Lee , S. E. How , Polymers 2021, 13, 3290. 34641106 10.3390/polym13193290 PMC8512023

[134] X. M. Peng , X. Liu , Y. Q. Yang , M. W. Yu , Z. W. Sun , X. R. Chen , K. Q. Hu , J. Yang , S. T. Xiong , B. Wang , L. Y. Ma , Z. X. Wang , H. X. Cheng , C. C. Zhou , Int. J. Nanomed. 2023, 18, 6725. 10.2147/IJN.S433624 PMC10659149 38026526

[135] A. Papaioannou , E. Vasilaki , K. Loukelis , D. Papadogianni , M. Chatzinikolaidou , M. Vamvakaki , Biomater. Adv. 2024, 157, 213737. 38211506 10.1016/j.bioadv.2023.213737

[136] A. A. Sadek , M. Abd‐Elkareem , H. N. Abdelhamid , S. Moustafa , K. Hussein , Sci. Rep. 2023, 13, 5404. 37012344 10.1038/s41598-023-32487-7 PMC10070441

[137] G. Choe , S. Oh , J. M. Seok , S. A. Park , J. Y. Lee , Nanoscale 2019, 11, 23275. 31782460 10.1039/c9nr07643c

[138] A. Sharma , S. Gupta , T. S. Sampathkumar , R. S. Verma , Biomater. Adv. 2022, 134, 112587. 35525768 10.1016/j.msec.2021.112587

[139] Y. C. Shin , J. Kim , S. E. Kim , S. J. Song , S. Hong , J. W. Oh , J. Lee , J. C. Park , S. H. Hyon , D. W. Han , Regener. Biomater. 2017, 4, 159. 10.1093/rb/rbx001 PMC5516678 28740639

[140] E. S. Motiee , S. Karbasi , E. Bidram , M. Sheikholeslam , Int. J. Biol. Macromol. 2023, 247, 125593. 37406897 10.1016/j.ijbiomac.2023.125593

[141] A. Aidun , A. S. Firoozabady , M. Moharrami , A. Ahmadi , N. Haghighipour , S. Bonakdar , S. Faghihi , Artif. Organs 2019, 43, E264. 31013365 10.1111/aor.13474

[142] S. Bahrami , N. Baheiraei , M. Shahrezaee , Sci. Rep. 2021, 11, 16783. 34408206 10.1038/s41598-021-96271-1 PMC8373942

[143] S. K. Liu , C. C. Zhou , S. Mou , J. L. Li , M. R. Zhou , Y. Y. Zeng , C. Luo , J. M. Sun , Z. X. Wang , W. H. Xu , Mater. Sci. Eng., C 2019, 105, 110137. 10.1016/j.msec.2019.110137 31546424

[144] P. N. B. Rebecca , D. Durgalakshmi , S. Balakumar , R. A. Rakkesh , J. Mater. Res. 2023, 38, 4314. 10.1016/j.chemosphere.2023.140273 37758069

[145] A. K. Mahanta , D. K. Patel , P. Maiti , ACS Biomater. Sci. Eng. 2019, 5, 5139. 33455220 10.1021/acsbiomaterials.9b00829

[146] J. N. Wu , A. Zheng , Y. Liu , D. L. Jiao , D. L. Zeng , X. Wang , L. Y. Cao , X. Q. Jiang , Int. J. Nanomed. 2019, 14, 733. 10.2147/IJN.S187664 PMC6342216 30705589

[147] Z. W. Xu , C. Y. Wang , G. Q. Song , Y. Wang , X. Y. Zhang , X. M. Li , Int. J. Biol. Macromol. 2023, 237, 124077. 36934820 10.1016/j.ijbiomac.2023.124077

[148] S. Wang , Y. Tao , Int. J. Polym. Mater. Polym. Biomater. 2023, 72, 1434.

[149] V. Chopra , J. Thomas , S. Kaushik , S. Rajput , R. Guha , B. Mondal , S. Naskar , D. Mandal , G. Chauhan , N. Chattopadhyay , D. Ghosh , Small 2023, 19, 2204637. 10.1002/smll.202204637 36642859

[150] X. Y. Wang , K. Jiang , W. J. Zheng , Z. Z. Bai , S. Huang , Z. Y. Chu , H. M. Liu , L. Liu , Chem. Eng. J. 2024, 479, 147360.

[151] S. D. Purohit , R. Bhaskar , H. Singh , I. Yadav , M. K. Gupta , N. C. Mishra , Int. J. Biol. Macromol. 2019, 133, 592. 31004650 10.1016/j.ijbiomac.2019.04.113

[152] Y. H. Hou , W. G. Wang , P. Bartolo , Mater. Today Bio 2024, 24, 100886. 10.1016/j.mtbio.2023.100886 PMC10761775 38173865

[153] S. S. Zhu , L. Y. Yao , C. L. Pan , J. H. Tian , L. H. Li , B. H. Luo , C. R. Zhou , L. Lu , Compos. Sci. Technol. 2021, 208, 108763.

[154] Z. W. Zheng , Y. H. Chen , H. Hong , Y. Shen , Y. Wang , J. Sun , X. S. Wang , Adv. Healthcare Mater. 2021, 10, 2000631. 10.1002/adhm.202000631 33166076

